# Graphene patterning without plasma etching via SU-8 pattern peel-off

**DOI:** 10.1038/s41598-025-08895-2

**Published:** 2025-07-04

**Authors:** Maryam Riyahi, Gholam-Mohammad Parsanasab, Mohammad Sabaeian

**Affiliations:** 1 Department of Physics, Faculty of Science, Shahid Chamran University of Ahvaz, Ahvaz, 6135743135 Iran; 2 Integrated Photonics Laboratory, Faculty of Electrical Engineering, Shahid Beheshti University, Tehran, 1983963113 Iran

**Keywords:** Graphene, SU-8, Graphene patterning, Graphene removal, Wet etching, Mechanical removal, Lithography, Graphene, Polymers

## Abstract

Although graphene has made its way into many areas of science and technology, proper tools for patterning graphene are not available to all researchers. Therefore, any new patterning method is useful. This research investigates the patterning of graphene layers on ${{Si{O_2}} \mathord{\left/ {\vphantom {{Si{O_2}} {Si}}} \right. \kern-0pt} {Si}}$ substrates via the use of an SU-8 photoresist to produce micrometer-sized components such as electrodes. The new method is based on the sufficient adhesion of graphene to SU-8 after SU-8 cross-linking. First, SU-8 photolithography in the inverse form of the final pattern is carried out on the graphene layer. Then, both the SU-8 pattern and the graphene part attached to it are simultaneously removed, resulting in the final graphene pattern. This method can also be extended in a way that is compatible with imprint lithography, as its framework is described in this paper. In this way, the non-crosslinked SU-8 in the inverse pattern is transferred to the graphene layer via a premade stamp. This latter approach could benefit from the complete elimination of SU-8 effects, especially SU-8 contamination, from the final graphene pattern, as well as the simplicity of replication for mass production.

**Supplementary Information:**

The online version contains supplementary material available at 10.1038/s41598-025-08895-2.

## Introduction

Graphene, a two-dimensional allotrope of carbon with $S{P^2}$ hybridization, has a wide range of applications in research and industry, from improving anti-corrosion layers to using it in quantum technologies^1,2^. The fabrication of various graphene-based devices in fields such as electronics^3,4^photonics^5^agriculture^6^and biotechnology^7,8^requires graphene patterning. Although some graphene patterning methods, such as electron beam lithography (EBL) combined with reactive ion etching^9^ or helium ion lithography, are very precise with nanometer-scale resolution^10,11^they require state-of-the-art technologies that are not accessible in every laboratory. Therefore, each new patterning method could be valuable.

Photolithography with oxygen plasma etching is one of the simplest and most widely used techniques for graphene patterning^12,13^. One of the main challenges in this regard is the adhesion of the photoresist to graphene and the difficulty of completely removing it from the graphene layer, especially after exposure to oxygen plasma^14,15^. This work demonstrates that it is possible to employ SU-8 photoresist adhesion as a beneficial attribute for removing graphene and replacing it with the plasma etching step. Any negative effects of bombarding the graphene substrate with ions or free radicals, such as ion penetration into the substrate or changes in the roughness of the substrate’s surface, are also eliminated by omitting the bombarding step.

This paper presents a graphene patterning method using SU-8, a well-known epoxy-based negative photoresist. This method is based on the proper adhesion of graphene to SU-8 after the cross-linking step, which occurs under particular conditions. The patterning of the graphene layer on the ${{Si{O_2}} \mathord{\left/ {\vphantom {{Si{O_2}} {Si}}} \right. \kern-0pt} {Si}}$ substrate starts with the photolithography of SU-8 in the inverse form of the desired pattern. Then, by peeling off the SU-8 scheme, the part of the graphene that adheres to the cross-linked SU-8 is simultaneously removed, and the final pattern of graphene remains. Mechanical removal is used to peel off cross-linked SU-8. A framework for the developed version of this patterning method is presented in the last section.

The adhesion of SU-8 has already been used for achieving ideal contact between graphene and the substrate^16,17^. However, to the best of our knowledge, no reports on graphene removal and patterning using peeling off SU-8 photoresist have been published. Generally, graphene patterning by removing another material due to its adhesion has been less reported. In many graphene patterning methods, graphene is removed via plasma or ion bombardment^18–21^. Dimiev et al. reported graphene patterning by sputtering zinc (or aluminum) on the graphene layer in the inverse of the desired pattern and then removing the zinc and its attached graphene into a diluted acid solution^22^. In this way, only one layer of graphene is removed in each process. Therefore, in the positive view, control of the final graphene layer is achieved, but in the negative view, to remove two-layer graphene, this process must be repeated, which requires expensive instruments such as a mask aligner. This method introduces the problem of residual resist as a contaminant in the final graphene pattern, as Dimiev et al. reported. In another paper, Alonso et al. reported a lithography method compatible with roll-to-roll manufacturing technology, all in a few brief sentences, while they were fabricating touch sensors on textile fibres^23^. They applied a prepatterned photoresist on graphene, which was subsequently removed with acetone. They reported that the removal of the photoresist resulted in an effective transfer of the pattern to the graphene layer, without any further discussion about this method or its advantages and disadvantages. Also, in some soft lithography methods, prepatterned PDMS was used to pattern a layer and simultaneously transfer the pattern. However, these techniques are often applied for coatings made from graphene oxide sheet solutions, reduced graphene oxide sheet solutions, or graphene sheet solutions^20,24,25^. The sheets adhere to the PDMS and transfer to a substrate to produce the desired pattern^26^. Although soft lithography methods are resist-free, some other materials, such as dimethyl sulfoxide, which is used to enhance the adhesion of PDMS, may remain on the final graphene pattern as contaminants. The low resolution of this method is reported in the literature, but the quality of the fabricated boundaries is not discussed^11,20^.

## Graphene patterning

**Fig. 1:**
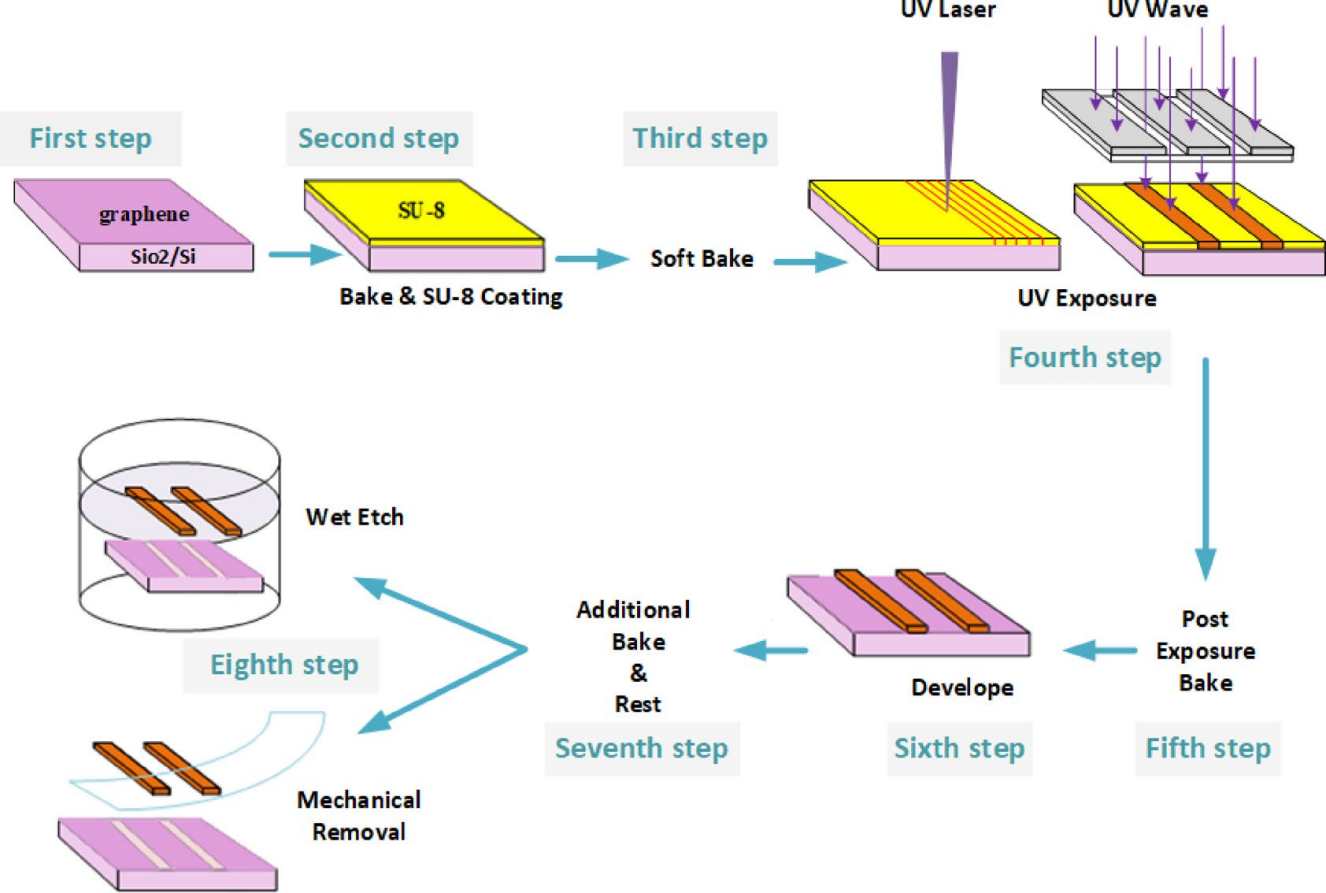
Schematic illustration of the SU-8-based graphene patterning process.

The steps of graphene patterning via SU-8 are schematically shown in (Fig. 1). The graphene layer synthesized via chemical vapor deposition (CVD) has been transferred to the ${{Si{O_2}} \mathord{\left/ {\vphantom {{Si{O_2}} {Si}}} \right. \kern-0pt} {Si}}$ wafer using the wet transfer method (first step)^27^. The standard SU-8 photolithography procedures are demonstrated in steps two through six. These include coating SU-8 onto a graphene layer with a spin coater, a soft bake (SB), UV exposure, post exposure bake (PEB), and a development step, respectively. These steps are explained in detail in the method section; only two important notes are mentioned here.

In the second step, the following five thicknesses of SU-8 (KayakuAM- MicroChem^28^) coating were examined for the lithography process: $0.6\mu m$ thickness of SU-8 $2000.5$, $2\mu m$ thickness of SU-8 2002, $5\mu m$ thickness of SU-8 2005, and$10\mu m$ and $25\mu m$ thicknesses of SU-8 2010. These tests revealed that SU-8 coatings with a maximum thickness of $5\mu m$ were appropriate for this lithography method. For a thicker coating, immersion in the developer for longer periods of time while being stirred can damage the graphene layer.

One of the important conditions leading to proper graphene adhesion to SU-8 is sufficient UV exposure in the fourth step. For example, in a $600nm$-thick SU-8 coating, a UV exposure energy greater than $60{{mj} \mathord{\left/ {\vphantom {{mj} {{{(cm)}^2}}}} \right. \kern-0pt} {{{(cm)}^2}}}$ is adequate for successful SU-8 lithography. However, the minimum exposure energy required for sufficient adhesion of graphene to SU-8 was determined to be $70{{mj} \mathord{\left/ {\vphantom {{mj} {{{(cm)}^2}}}} \right. \kern-0pt} {{{(cm)}^2}}}$.

After SU-8 lithography on the graphene layer, further baking at ${95^ \circ }C$ is performed in the seventh step. Following a few minutes of cooling, the SU-8 pattern and its adhesive graphene are peeled off, and the graphene pattern appears (the eighth step).

**Fig. 2:**
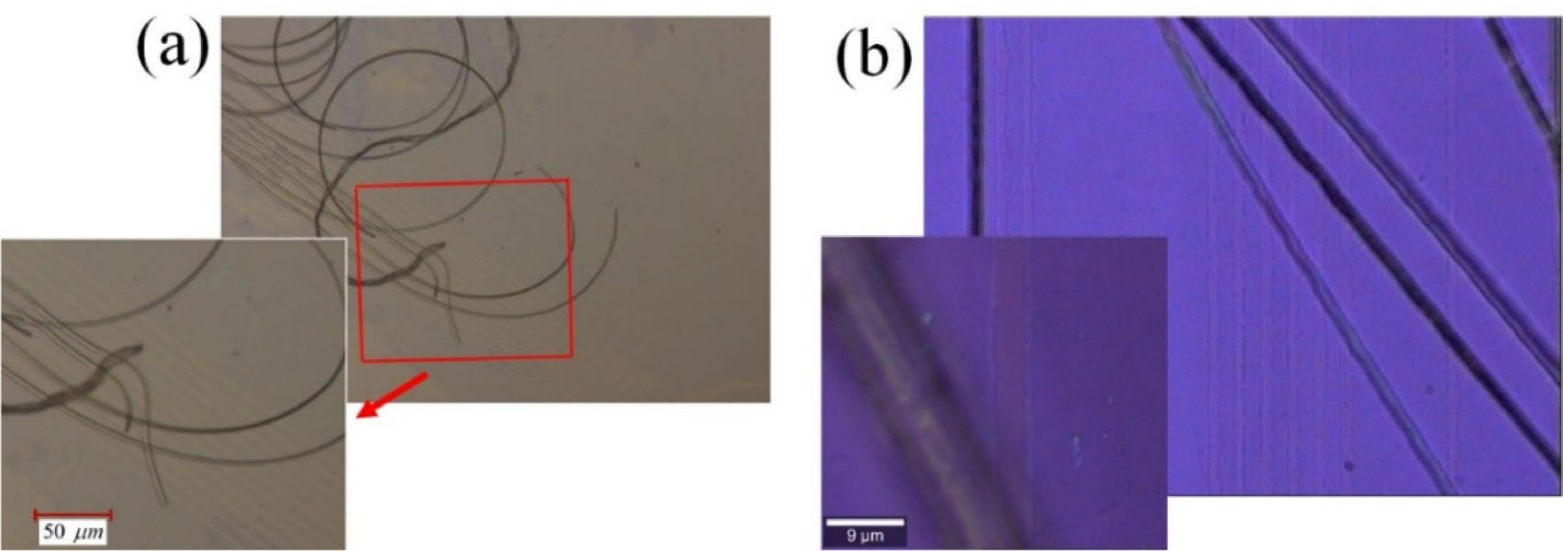
The channels are fabricated on the graphene layer with a resolution finer than $5\mu m$, while the SU-8 strips and their adhesive graphene parts are being peeled off during the wet etching process (**a**) and the mechanical removal process (**b**). The initial SU-8 strips had a $2\mu m$ thickness in (**a**) and a $5\mu m$ thickness in (**b**). The image in (**a**) was recorded with an optical microscope from Leica, and the image in (**b**) was taken with a microscope from WiTec.

In the initial attempt, a wet etching process utilizing PG Remover^29^ was employed to remove the cross-linked SU-8 pattern along with its adhesive graphene pattern (see supplementary information 1 (S1)). Typically, wet etching of SU-8 results in peeling rather than complete dissolution of the SU-8 pattern^29^. Figure 2(a) shows a microscopy image of the SU-8 strips during peeling off while creating channels in the graphene layer. During this process, especially in samples with extensive SU-8 patterns, certain areas may detach earlier than others. These detached regions can be carried away by the solvent flow, potentially colliding with and damaging the graphene layer. Additionally, detached SU-8 fragments may redeposit onto the sample surface, causing contamination. These issues are not encountered in the mechanical removal method. In the mechanical removal method, a piece of Scotch tape is placed over the SU-8 pattern, and upon peeling the tape off, both the SU-8 pattern and all graphene sections adhered to it are removed. The microscopic images in Fig. 2(b) show the SU-8 strips being peeled off from the substrate using Scotch tape (from Graphene Supermarket^30^) to create channels in the graphene layer. The SU-8 pattern is easier to remove mechanically, particularly when the SU-8 elements are thick, closely spaced, and interconnected. As a result, the graphene layer in most samples in this study was removed using the mechanical removal method.

## Experimental results and verification of graphene removal

**Fig. 3:**
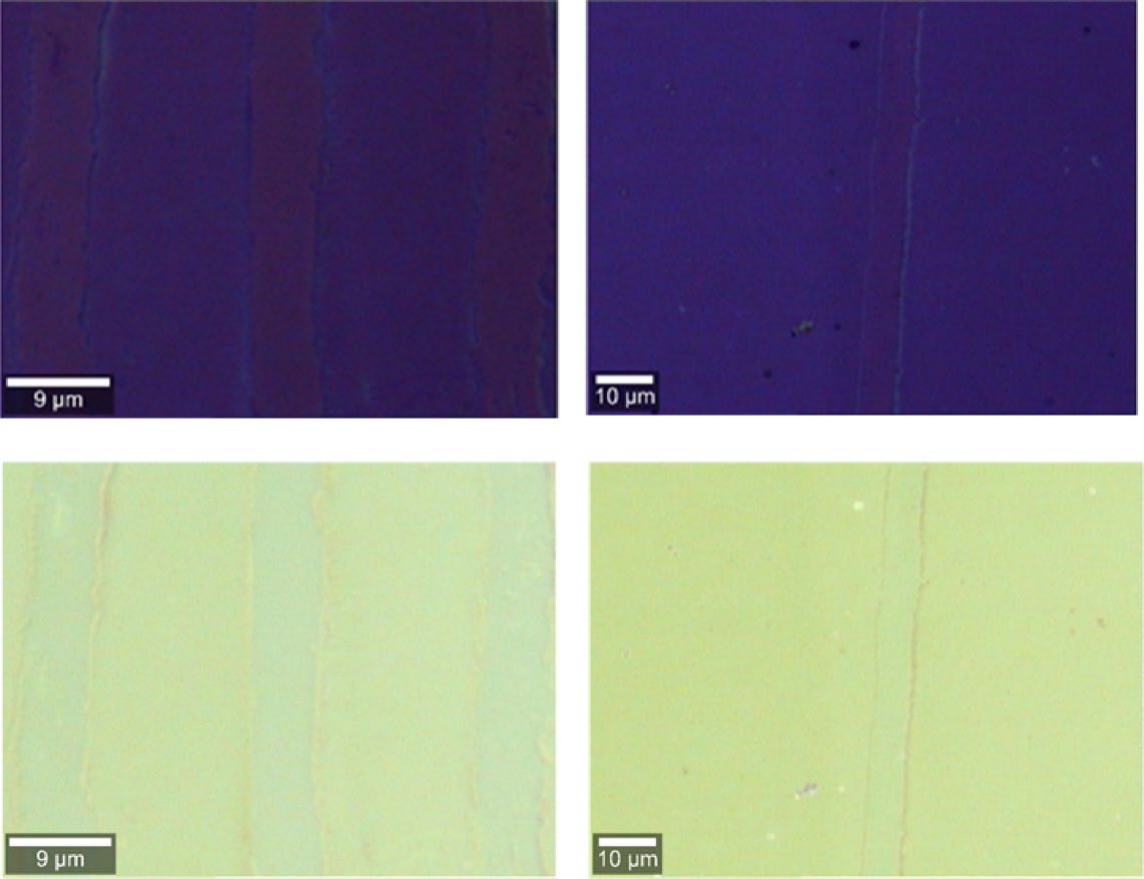
Optical microscopy images of the graphene strips and channels on the ${{Si{O_2}} \mathord{\left/ {\vphantom {{Si{O_2}} {Si}}} \right. \kern-0pt} {Si}}$ wafer are shown in the top row. These channels were formed via lithography of the SU-8 strips on the graphene layer followed by their mechanical removal. Owing to the low optical contrast between the graphene and substrate, the channels and strips may be difficult to see in the top row, so each image is filled with green and displayed in the bottom row.

After the graphene pattern on the ${{Si{O_2}} \mathord{\left/ {\vphantom {{Si{O_2}} {Si}}} \right. \kern-0pt} {Si}}$ wafer was fabricated, the results were analyzed and validated via optical microscopy, Raman spectroscopy, and atomic force microscopy (AFM). Figure 3 displays two microscopy images of the strips and channels formed in the graphene layer, with measured channel widths of $6\mu m$ and $7\mu m$.

**Fig. 4:**
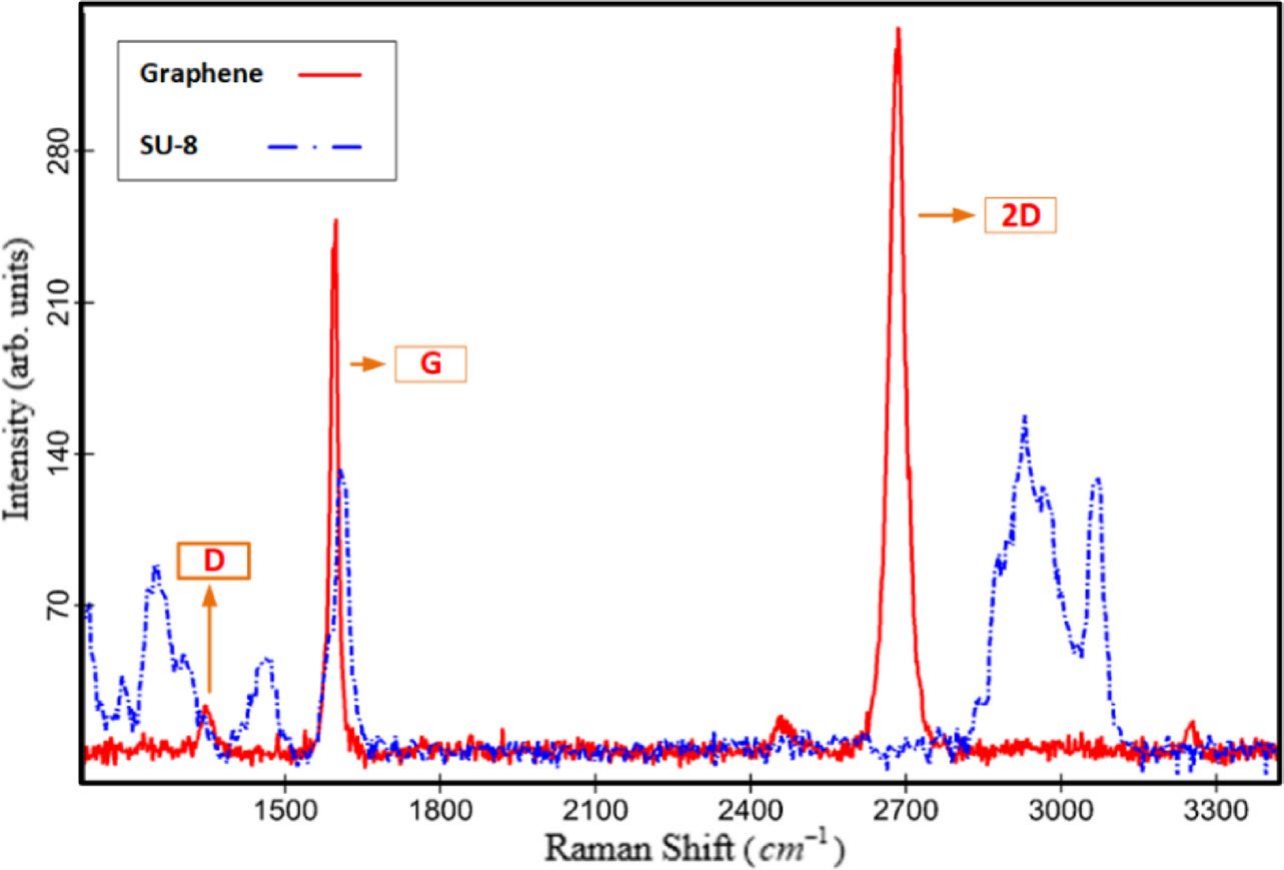
Raman spectra of graphene (red solid) and SU-8 (blue dashed) at key intervals, The *G* and *D* peaks of graphene overlap with some peaks of SU-8.

The Raman spectrum of graphene, like those of other carbon materials, has two prominent peaks: the $2D$ peak around $2700c{m^{ - 1}}$ and the *G* peak around $1582c{m^{ - 1}}$^31–33^ (Fig. 4). If there is a high level of crystalline order in the graphene sample, the *D* peak (around $1350c{m^{ - 1}}$) will be absent^31,34^. In fact, the *D* peak is the result of symmetry breaking at the graphene caused by defects such as $s{p^3}$ hybridization and vacancies, as well as the presence of substitutional atoms, edges, defected wrinkles, crystalline grain boundaries, and a folded layer^31,33,35–38^. Moreover, the $D^{\prime}$ peak (approximately $1620c{m^{ - 1}}$) appears when some of these agents increase. The graphene spectrum may also contain several low-intensity peaks, such as $D+D^{\prime\prime}$ peak around the $2450c{m^{ - 1}}$.

The characteristic Raman peaks of graphene can change with doping and strain^31,39^. In this study, evidence of doping was observed in the initial graphene samples on the ${{Si{O_2}} \mathord{\left/ {\vphantom {{Si{O_2}} {Si}}} \right. \kern-0pt} {Si}}$ substrates since the ${\omega _G}$ frequencies of their Raman spectra were measured around $1599C{m^{ - 1}}$, indicating an upshift from pristine monolayer graphene at $1580C{m^{ - 1}}$^40^. Additionally, the measured width of the $2D$ peaks in their Raman spectra, approximately $34C{m^{ - 1}}$, exceeded the value of $16C{m^{ - 1}}$ for defect-free, doping-free, and stress-free graphene (for a ${\lambda _{Laser}}=532nm$). This suggests the presence of strain in the initial sample, particularly the nanometer-scale strain variation on the length scales far below the laser spot size^41^. To distinguish between the contributions of doping and strain, additional control over the Raman measurement is needed, which was not our concern in this study^16^.

Figure 4 shows the Raman spectra of one of the graphene samples and the SU-8 coating separately on the ${{Si{O_2}} \mathord{\left/ {\vphantom {{Si{O_2}} {Si}}} \right. \kern-0pt} {Si}}$ substrate (see supplementary information S2). The intensity of the graphene peaks is greater than that of the SU-8 peaks, making the *D* peak more visible. A comparison of the Raman spectra of graphene and SU-8 reveals that some SU-8 peaks overlap with the graphene *G* peak, especially on its left shoulder, as well as with potential graphene $D{,^{}}D^{\prime}$ and $D+D^{\prime}$ (approximately $2970c{m^{ - 1}}$) peaks. Therefore, the visibility and analysis of the Raman spectra of graphene samples beneath a thick SU-8 coating are challenging (see supplementary information S3).

**Fig. 5:**
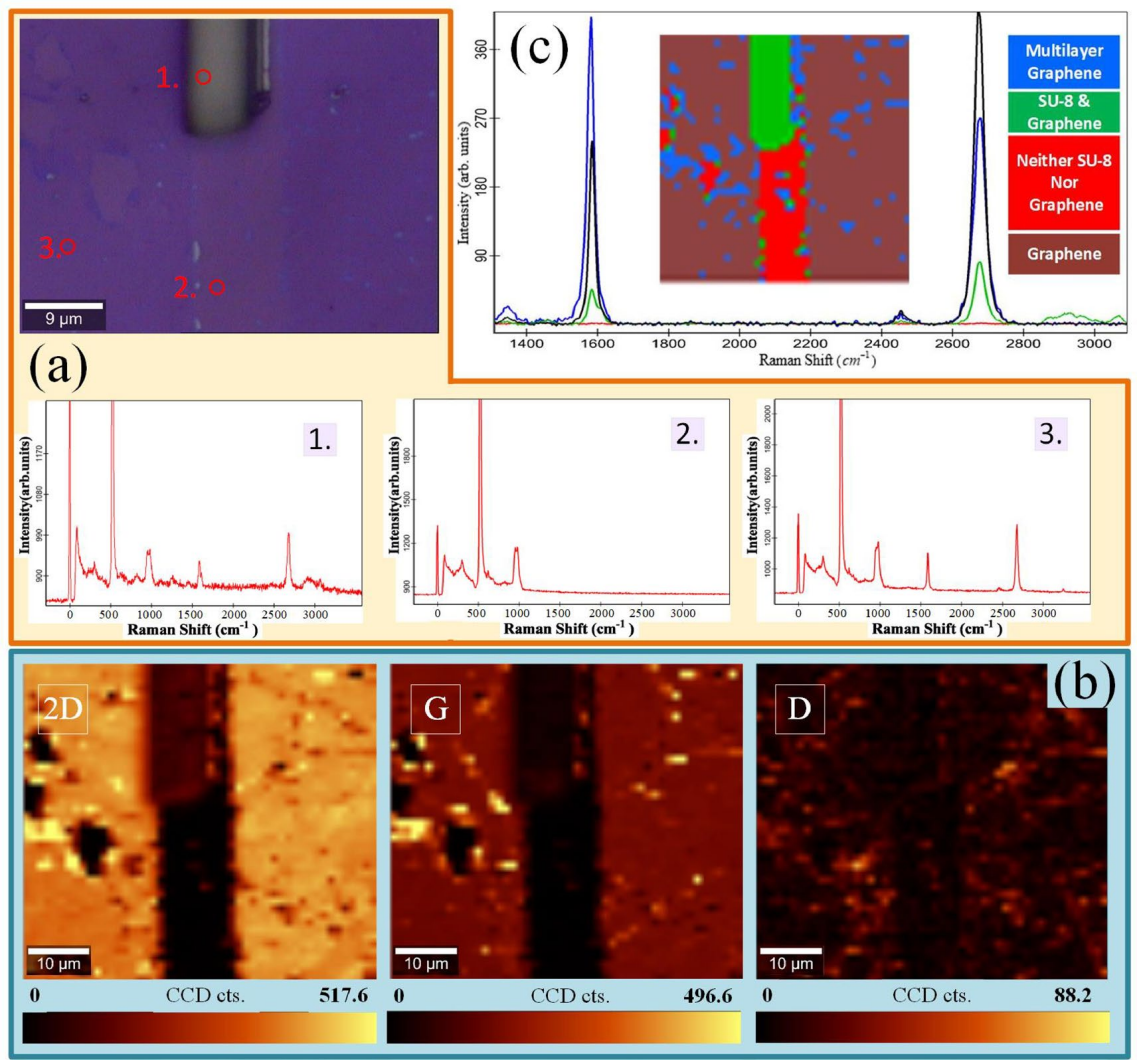
(**a**) A microscopy image of the cross-linked SU-8 strip created by photolithography on the graphene layer. The SU-8 strip has been separated in part, creating a channel in the graphene layer. Additionally, single Raman spectra at arbitrary points in three different regions, at the center of the red circles, are shown. (**b**) These images show the 2D, G, and D Raman intensity maps of this sample. The horizontal color bars show how the peak intensity decreases as the darkness increases. In the color bar, two numerical bounds are selected to provide the greatest possible contrast in the image. (**c**) On the basis of the similar average Raman spectra, the sample was divided into four different areas. Each area and its corresponding average Raman spectrum have the same color. Because of the presence of the SU-8 strip, any other spot in the sample exhibiting similar peaks, even at very low intensity, will be classified as SU-8, indicating SU-8 contamination.

Figure 5(a) shows a microscopy image of a sample in which, in the final steps of the patterning process, part of its SU-8 strip has not been removed for more clarity. This image displays three obvious regions: the first region is a part of the SU-8 strip on the graphene layer, the second region is a channel along the SU-8 strip from which SU-8 and graphene have been removed, and the third region is the remaining graphene layer. The single Raman spectra in (Fig. 5(a)), which were measured at an arbitrary point (at the center of the red circles), and the Raman maps of the $2D$, *G*, and *D* peaks over the surface of the sample in (Fig. 5(b)) confirm the presence of these regions. In particular, the Raman spectrum of the second point verifies the absence of any characteristic peaks of graphene or SU-8 (Fig. 5(a)). Additionally, the $2D$ and *G* peak intensities across the channel are almost zero  (Fig. 5(b)), which shows the absence of graphene in the channel.

From another point of view, this sample is divided into four regions based on similar average Raman spectra, although with very different intensities (see the inset of Fig. 5(c)). The four areas include the three previously mentioned regions and the scattered small regions of multilayer graphene. A comparison of the Raman spectra is easier in this figure. The red spectrum does not have any peaks and is related to the red channel without any graphene or SU-8. The brown spectrum corresponds to graphene without SU-8. The blue spectrum has a more intense *G* peak than its $2D$ peak, and it also has a *D* peak. Therefore, the blue areas belong to multilayer graphene with some defects. The green spectrum shows the characteristic peaks of SU-8 and graphene simultaneously. Therefore, the green area shows the SU-8 strip and the green spots caused by SU-8 contamination. Note that the intensity of the SU-8 peaks at these contamination spots is very low and only slightly above the noise. A detailed comparison of the variations in intensity of the SU-8 Raman peak at various contamination locations and the SU-8 strip is available in the supplementary S4.

**Fig. 6:**
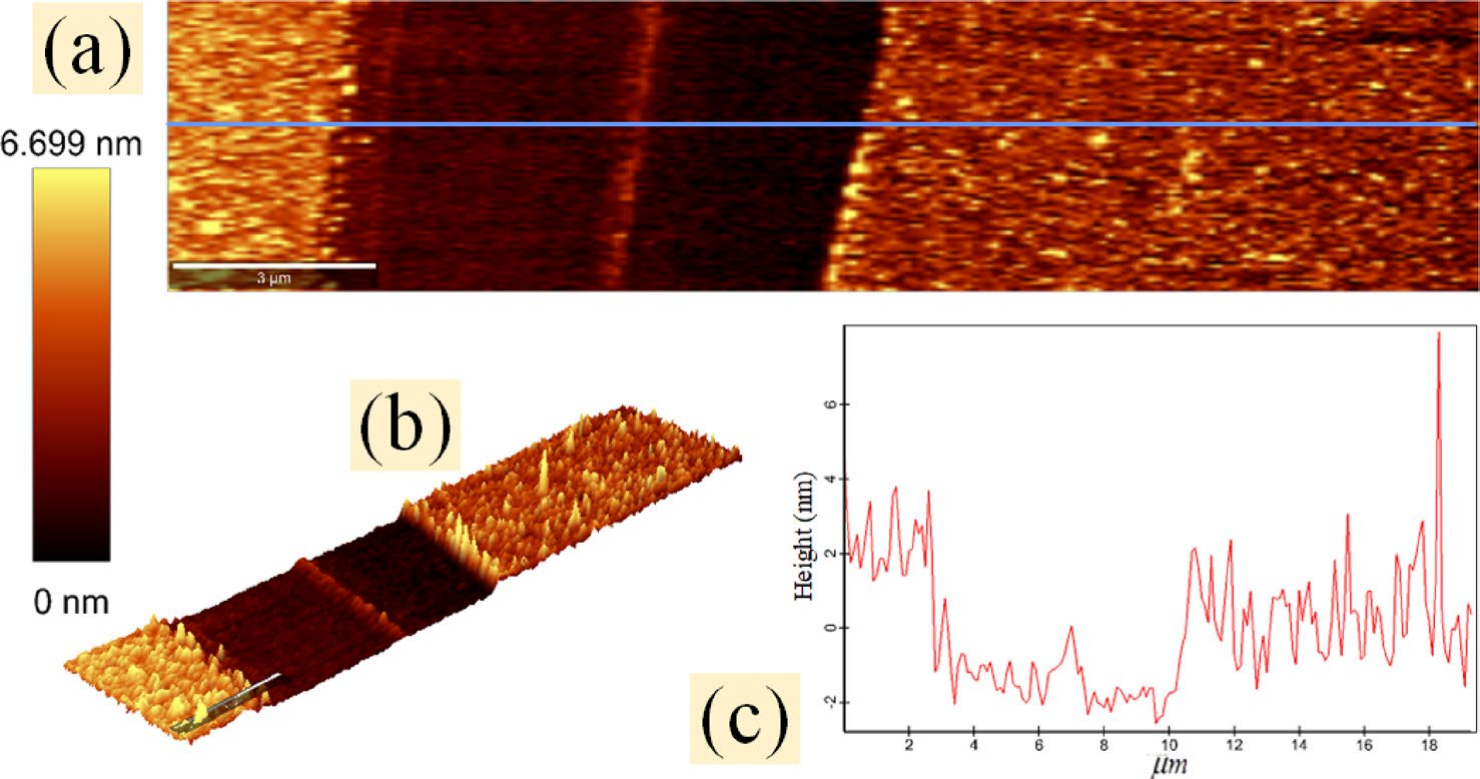
(**a**,**b**) AFM topography images of the graphene layer and its channel in two-dimensional view and three-dimensional vision, respectively. (**c**) The cross-section of the topography image along the blue line clearly shows the height variations within and surrounding the channel.

The AFM topography image is another reason for graphene removal, as evidenced by the height difference between the remaining graphene layer and the removed graphene region. The height differences are shown with topography images in two- and three-dimensional views in Figs. 6(a) and 6(b), respectively. Figure 6(c) displays the cross section of the topography image along the blue longitudinal line.

According to the results of this section, the removal and patterning of graphene using SU-8 were successfully verified. The graphene domains on the samples in this study included monolayer, bilayer, and small portions of multilayer graphene (see supplementary information S5). SU-8 effectively removed one- and two-layer graphene sheets. However, multilayer graphene remained as contamination after the process (visible as blue areas in the red channel of (Fig. 5(c)).

## Discussion

The SU-8 photoresist (SU-8 2000 from KayakuAM) contains three ingredients:$EPO{N^{TM}}$ SU-8 resin, the photoacid generator triarylsulfonium hexafluoroantimonate salt, and cyclopentanone^28,42^. Triarylsulfonium hexafluoroantimonate, in the presence of UV radiation, generates free radicals, especially ${H^+}$ions, which are strong Lewis acids^42–44^. The ${H^+}$ ion acts as a catalyst for the cross-linking process at the baking step by opening the epoxy ring of the SU-8 molecule^45,46^. Because of its low baking temperature in this work, only the SU-8 crosslinking process occurs, and there is no possibility of SU-8 carbonization^47,48^. The adhesive properties of epoxy-based materials improve with opened epoxy rings because of increased polarizability^49^especially for the SU-8 molecule with eight functional groups (see supplementary information S6). In this manner, Wang et al. reported polymer grafting onto the surface of SU-8 during UV exposure^42^. In total, free radicals and opened epoxies of SU-8 during UV exposure could increase the interaction and adhesion of SU-8 to its adjacent materials, which, in this study, is the graphene layer^50,51^. In the following paragraphs, from a different perspective, some possible agents in the graphene layer for interaction with SU-8 during the lithography process are considered.

Small atomic defects or functional groups in graphene enhance its interaction and adhesion to polymer, which has already been proven for graphene and PMMA^52^. This is why most of the residual SU-8 contamination in our samples was observed at the graphene edges or multilayer graphene spots (Fig. 5(c)). Additionally, it has been reported that the reactivity of graphene at grain boundaries or defects increases by mechanical strain, which appears during SU-8 coating or different baking steps^53^.

In the most obvious interaction between graphene and SU-8, correlated charge fluctuations of SU-8 molecules and inhomogeneous $\pi$-conjugation on the supported graphene layer cause van der Waals interactions^49^. Charge inhomogeneity and $\pi$ bond variation in the supported graphene layer, because of its ripples and curvatures, have already been reported^34,40,54–56^. The van der Waals interaction also becomes stronger during SU-8 crosslinking through the creation of hydroxyl groups and free radicals with greater polarizability and reactivity^46,49^. For example, hydroxyl groups in SU-8 and $\pi$ bonds in graphene could cause $OH - \pi$interactions^57^. Additionally, Lewis acids in SU-8 and $\pi$ bonds in graphene could cause $H - \pi$ interactions, a type of hydrogen bonding^56^. Furthermore, the SU-8 molecule has aromatic rings, which could cause $\pi - \pi$ interactions with graphene^56^. Owing to the extended $\pi$ orbitals on graphene, van der Waals interactions should be important factors for adhesion of SU-8 and graphene layers, especially in the absence of defects and associated covalent bonds. An investigation of the Raman spectra of graphene during the lithography process demonstrated the importance of the noncovalent interactions between graphene and SU-8. Raman spectra were measured at some random points on graphene far from the obvious microscopic defects during the lithography process (see supplementary information S7).

**Fig. 7:**
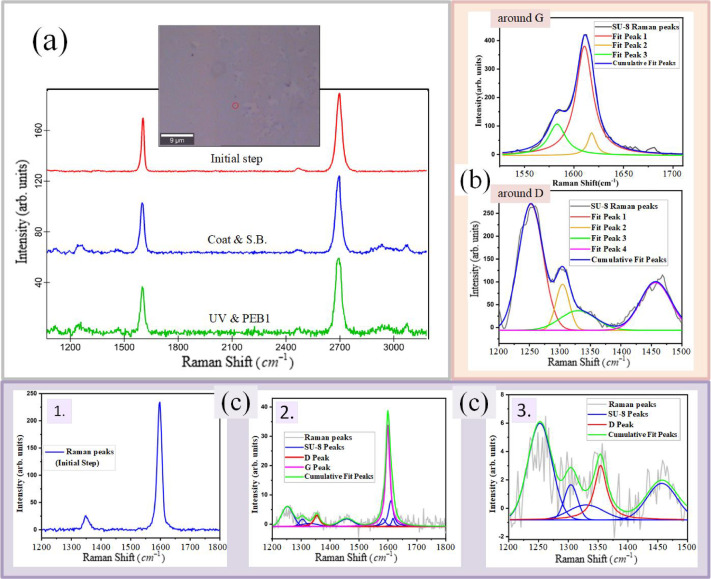
(**a**) A microscopy image of the first selected point, along with its Raman spectra at the initial step, before SU-8 coating and after several steps of the lithography process, is shown according to their labels. The SU-8 peaks have a low intensity because of the thin (600 nm) SU-8 coating. (**b**) The SU-8 spectrum (see supplementary information S2) on the ${{Si{O_2}} \mathord{\left/ {\vphantom {{Si{O_2}} {Si}}} \right. \kern-0pt} {Si}}$ substrate **around** *G* (**around** *D*) has been fitted by three Lorentzian (four Gaussian) subpeaks. (**c**) The Raman spectrum of the second selected point at the initial step shows a small *D* peak (**c-1**). The Raman spectrum of this selected point after UV exposure and PEB at the equivalent frequency was fitted with SU-8 subpeaks and the *G *and *D* peaks of graphene (**c-2**). For clarity, the frequency interval from ${1200_{}}C{m^{ - 1}}$ to ${1500_{}}C{m^{ - 1}}$ is shown in the last figure. The red peak shows the pure *D* peak of graphene (**c-3**).

The microscopy images and Raman measurements were investigated at some random points of graphene on ${{Si{O_2}} \mathord{\left/ {\vphantom {{Si{O_2}} {Si}}} \right. \kern-0pt} {Si}}$, before processing and after SU-8 coating and SB, as well as following UV exposure and PEB. Figure 7(a) shows the first selected point and its corresponding spectra. For this investigation, a $600nm$ SU-8 (2000.5) coating was used to reduce the intensity of the SU-8 peaks in the Raman spectra of the samples. To accurately find the *G* and *D* peaks of graphene at these selected points, the SU-8 Raman peaks around the *G* and *D* peak frequencies were matched with some subpeaks (Fig. 7(b)) and subtracted from the Raman spectra of the selected points (supplementary information S8, S9 and Fig. 7(c)).

This investigation revealed that the Raman spectra of the selected points, which did not display a visible *D* peak in the initial steps, continued to show no *D* peak after the $600nm$ SU-8 coating and subsequent steps of the lithography process (supplementary information S9 and (Fig. 7(a)). Therefore, there is no increase in defects or $S{P^3}$ bonds at these selected points during the lithography process, nor is there a change in graphene hybridization. At some other selected points, there was a low-intensity *D* peak in the initial step. Even at these selected points, the increase in the *D* peak intensity after the process was very low. The spectral region corresponding to the second selected point, both before and after the process, is shown in (Fig. 7(c)). In this case, the ratio of ${{{I_D}} \mathord{\left/ {\vphantom {{{I_D}} {{I_G}}}} \right. \kern-0pt} {{I_G}}}$ increases from $0.103$ to $0.111$, which is low. These results highlight the importance of noncovalent bonding in graphene-SU-8 adhesion at these selected points.

After crosslinking, the majority of points in all samples with varying thicknesses of the SU-8 coating, including the selected points from the final tests, exhibited ${{{I_{2D}}} \mathord{\left/ {\vphantom {{{I_{2D}}} {{I_G}}}} \right. \kern-0pt} {{I_G}}}>1$ (see supplementary information S3). Therefore, the adhesive layer on SU-8 remains graphene at all steps of the process. SU-8 patterns that peel off with adhesive graphene may be useful in some technologies, such as microelectromechanical systems (MEMS).

The latest discussion is about the limitation of the graphene patterning method using SU-8, which is the edge quality of the final graphene pattern. A comparison of AFM results across samples shows that graphene surface roughness remains largely unchanged before and after patterning graphene, except at the edges of the final graphene pattern (Fig. 6(c) and S10). A pristine graphene layer has excellent mechanical strength and elastic modulus^34,35^. The average elastic moduli of 0.91$TPa$ and 1$TPa$ have been reported for CVD graphene and mechanically exfoliated graphene, respectively^35,58^. Although the breaking load of graphene decreases with an increasing number of defects or functional groups^35^, at the sixth step of the lithography process in our samples, the graphene layer in the two areas, especially at its boundaries, has good fracture strength. (Two areas are defined for the graphene layer on the sample: the “first area” of graphene, which is covered with crosslinked SU-8 at the sixth step of the lithography process (Fig. 1), and the “second area,” which includes the remaining graphene layer.) Therefore, when the SU-8 pattern and the first area of graphene are peeled off, the boundary between the two areas ruptures after enduring considerable tension. Because of this tension, the edges of the second area (final graphene pattern) in our samples were somewhat separated from the substrate, often by a width of 1 to 2 micrometers and a height ranging from zero to fifty nanometers (see supplementary information S10).

The process of cross-linking SU-8 proposes that extra baking in the seventh step of the lithography process (Fig. 1) can moderate the graphene edge issue. During UV exposure and PEB, SU-8 starts to cross-link when the temperature exceeds ${T_g}$ (the glass transition temperature). This process causes its linear oligomers to convert to a cross-linked network, resulting in SU-8 shrinking^43,44^. SU-8 shrinking exerts compressive strain on its underlying graphene (the first area of graphene). Therefore, the bonds of the carbon atoms in the boundary between two areas are weaker, the tension during the tear of the boundary is lower, and the final height of the graphene edge is also lower. According to this explanation, doubling the baking time by adding a seventh step of the lithography process leads to a better edge quality of the final graphene pattern. This explains why a nanometer graphene strip, approximately 350 nm wide, forms in the middle of the channel in certain samples (Fig. 6). Prior to any processing, the ${T_g}$ of SU-8 is approximately ${50^ \circ }C$. It increases with additional baking and cross-linking^43,44,59^but it takes more than 2 minutes to reach ${95^ \circ }C$^43^. Therefore, in the seventh step of the lithography process, the baking temperature is still higher than ${T_g}$, and SU-8 cross-linking continues to occur. If the time interval between the fourth and seventh steps of the lithography process (Fig. 1) is long, the sample is exposed to UV radiation before baking in the seventh step. During sample cooling, the strain on the first area of the graphene from SU-8 or $Si{O_2}$ is compressive^34,43^.

As a technical note, the negative effect of manual peeling of SU-8 on graphene edge quality is removed by using a stage to control the motion of the Scotch tape . Additionally, the contamination-induced height at the edge is reduced by performing the procedure in a cleanroom. (see supplementary information S10)

For certain elements, such as micrometer-scale electrodes, the nanometer-scale separation of the edge from the substrate can be ignored. Therefore, graphene patterning via the mechanical removal of SU-8 is a way to fabricate micrometer-sized graphene elements, such as electrodes. Finally, it is worth noting that chemists may be able to optimize the SU-8 formulation by adjusting the ratio of Lewis salts and other components for this application. During SU-8 crosslinking, this could facilitate covalent bonding between SU-8 and graphene, similar to the bonding observed with diazonium salt treatment^60^. These covalent bonds induce sp³ hybridization and create vacancies in the graphene areas beneath the SU-8. As a result, the continuity of carbon–carbon bonding at the boundary between the SU-8-adhered and free graphene (two areas of graphene) is disrupted, leading to a desirable improvement in edge quality.

## Patterning graphene using SU-8 in a way compatible with imprint lithography

With a small modification, the method of patterning graphene via SU-8 can be adapted into a new technique that is compatible with imprint lithography. This section presents the idea and framework for this new approach.

**Fig. 8:**
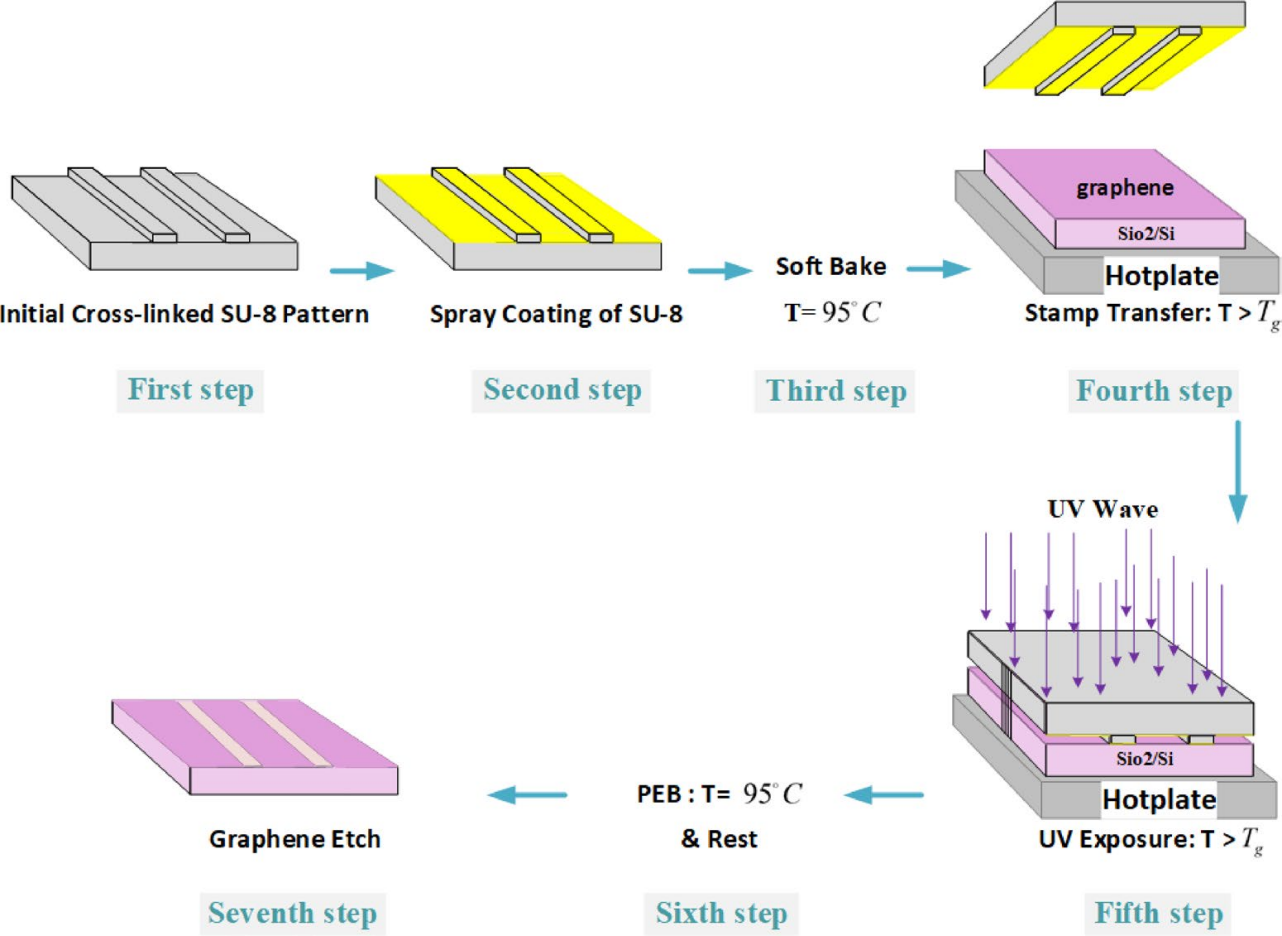
Schematic illustration of the SU-8-based patterning of graphene, which is compatible with imprint lithography. The first three steps show stamp fabrication.

Figure 8 shows the basic idea of the process. In the first step, an inverse version of the desired pattern will be fabricated on an $10\mu m$SU-8 layer coated on the substrate. A proper substrate must have good transparency at $360nm$ and good adhesion with SU-8. For example, glass covered with a thin layer of $A{l_2}{O_3}$ could be used as a substrate. The first step includes $10\mu m$SU-8 spin-coating on the substrate, a soft bake at ${95^ \circ }C$, UV exposure, PEB at ${95^ \circ }C$, and a final development step to produce the SU-8 pattern^28^. After that, 2$\mu m$ of SU-8 are coated on the fabricated structure via the spray coating technique to preserve the previously created pattern (Fig. 8, second step)^61^. In the third step, a soft bake is performed at ${95^ \circ }C$ to evaporate some of the solvent. In fact, these three steps create a proper stamp for transferring the inverse pattern to the graphene layer.

The fourth step involves heating both the stamp and the graphene layer on the ${{Si{O_2}} \mathord{\left/ {\vphantom {{Si{O_2}} {Si}}} \right. \kern-0pt} {Si}}$ substrate to a temperature of ${T_g}$, transferring the stamp onto the graphene sample, and pressing the stamp on the graphene as the hotplate temperature rises above ${T_g}$. At the optimal temperature and pressure, full contact between the uncrosslinked SU-8 and the graphene layer takes place in the inverse pattern. In the fifth step, the stamp will expose the sample to UV light under these conditions. After that, a PEB step raises the temperature to ${95^ \circ }C$. During the cross-linking of the $2\mu m$ SU-8 layer, both its adhesion to the graphene layer and its bonding to the initial 10$\mu m$ SU-8 layer increase. These steps are the same as the process already reported for the fabrication of free-standing SU-8 microfluidic chips by adhesive bonding^62,63^. After PEB, the sample is left to cool for a few minutes. Finally, in the seventh step, the stamp is separated from the graphene layer, and the final pattern is expected to emerge.

The basis of this method and the reasons for graphene adhesion to SU-8 are the same as those in the previous process. The only difference in this method is the way contact is established between non-crosslinked SU-8 and graphene. Sufficient contact using this approach has already been reported between uncrosslinked SU-8 and other layers, such as crosslinked SU-8 or glass^62,64^. Better contact between graphene and SU-8 in this approach can lead to a higher-quality final graphene pattern. Additionally, this technique eliminates photoresist-related effects, especially SU-8 contamination, from the final graphene pattern by omitting the SU-8 coating step on the graphene layer. This imprint lithography process could be a suitable method for some industrial production of graphene-based elements.

## Conclusion

In summary, this work presented a new technique for patterning graphene via cross-linked SU-8 to fabricate micrometer elements, such as electrodes. This method utilized photolithography to pattern SU-8 onto the graphene layer in an inverted version of the desired pattern. Afterward, the crosslinked SU-8 was peeled off, taking with it the graphene regions that adhered to it. These steps produced a graphene pattern on the ${{Si{O_2}} \mathord{\left/ {\vphantom {{Si{O_2}} {Si}}} \right. \kern-0pt} {Si}}$ substrate, which was confirmed by Raman spectroscopy and AFM measurements. Mechanical removal was used to peel off the SU-8 pattern and its adhesive graphene. The mechanical removal of a 5$\mu m$ SU-8 patterns utilizing Scotch tape was introduced as a simpler method.

The investigation of the Raman spectra at random graphene points during the lithography process, along with a review of the literature, emphasized the importance of noncovalent interactions in graphene adhesion to SU-8 far from the defects. Three techniques were used to improve the final graphene pattern edge: baking during the seventh step of the lithography process, using a stage to precisely control the movement of the Scotch tape when peeling off the SU-8 pattern, and performing the test in a cleanroom.

The method of graphene patterning via SU-8 can be developed in a manner compatible with imprint lithography, as its framework was discussed in the last section. One of the advantages of the latter approach is the complete removal of photoresist contamination from the graphene pattern. Additionally, this method may be considered in industrial mass production.

## Methods

### SU-8 photolithography procedures on the graphene layer

The initial CVD graphene layers were purchased from Graphenea Company^65^ and transferred to ${{Si{O_2}} \mathord{\left/ {\vphantom {{Si{O_2}} {Si}}} \right. \kern-0pt} {Si}}$ substrates via the wet transfer method^27^. The thickness of the $Si{O_2}$ in the substrates was $285nm$. In each step of the SU-8 lithography process from the KayakuAM Company (Fig. 1), the parameters corresponded to the datasheet^66^unless otherwise stated. Since the parameters are dependent on the SU-8 thickness, in the following steps, only the parameters corresponding to the $600nm$ SU-8 layer are expressed in parentheses^28^.

The SU-8 layer was coated on the initial sample in the second step of Fig. 1 (for the $600nm$ SU-8 coating, it was spun at 2000 rpm for 30 s). In the third step, soft baking was performed at ${95^ \circ }C$ using a hotplate ($600nm$SU-8: SB for one minute). After cooling, the sample was exposed to UV light in the inverse version of the desired pattern (the fourth step). In this work, a laser direct writer setup with a continuous-wave laser at $360nm$ was used ($600nm$SU-8: UV exposure energy of $70{{mj} \mathord{\left/ {\vphantom {{mj} {{{(cm)}^2}}}} \right. \kern-0pt} {{{(cm)}^2}}}$). This step can also be performed with the use of a UV lamp and an appropriate mask.

After UV exposure, the sample was transferred to a hotplate at ${95^ \circ }C$ for a post-exposure bake in the fifth step of Fig. 1 ($600nm$ SU-8: PEB for one minute). Any further baking after cross-linking must be avoided. During this step, the pattern of the cross-linked SU-8 was distinguished on the SU-8 layer. Afterward, the sample was left for a few minutes to cool gradually. In the sixth step, the sample was submerged in the SU-8 developer at room temperature and shaken to remove the uncrosslinked SU-8 ($600nm$ SU-8: immersion time of one minute)^67^. At this step, the pattern of SU-8, which is the inverse of the desired graphene pattern, appeared. In the seventh step, further baking at ${95^ \circ }C$ was carried out immediately after the sample dried ($600nm$ SU-8: baking for one minute). After the development step, the sample was immersed in 2-propanol, followed by a deionized water bath, to rinse off the SU-8 developer. For mechanical removal, Scotch tape from the Graphene Supermarket Company was used.

### Microscope imaging, Raman spectroscopy, and atomic force microscopy

A WITec alpha 300RAS confocal microscope (equipped with a 100$\mu m$ pinhole) was employed for all characterization procedures and tests, with the exception of the microscope imaging shown in (Fig. 2(a)). The room-temperature Raman spectra were measured using a $100X$ objective, a $532nm$ laser excitation wavelength, and a spectrometer with a grating of 600 grooves/mm (or 1200 grooves/mm in a few tests). To protect graphene and SU-8 from thermal degradation, the excitation laser power was tuned to be less than $1mW$ at the surface of the samples. However, for samples with a thinner SU-8 layer, such as $600nm$, the laser power was adjusted to 60$\mu W$. The AFM topographies were measured in AC mode.

To investigate the Raman measurements of the selected points on the graphene layer in the discussion section, the integration time was increased to 10 s, the laser spot size was $500nm$, and the laser power was only 60$\mu W$.

## Electronic supplementary material

Below is the link to the electronic supplementary material.

Supplementary Material

## Data Availability

Data is provided within the manuscript or supplementary information files.
