# Supplementary material for: Graphene patterning without plasma etching via SU-8 pattern peel-off
Source: Sci Rep. 2025 Jul 4;15:23910. doi: 10.1038/s41598-025-08895-2 (PMC12227667; doi:10.1038/s41598-025-08895-2)
Supplement: Supplementary file 1 — Supplementary Material [file 41598_2025_8895_MOESM1_ESM.pdf]

Supplementary Information:

The Possibility of Graphene Patterning by Peeling the SU-8 Pattern Off

Maryam Riyahi, Gholam-mohammad Parsanasab, Mohammad Sabaeian*

S1:

The more cross-linked SU-8 is more difficult to remove. Several techniques, such as water jetting, ozone solutions, and O_2/fluorine plasma, have been suggested for cross-linking SU-8 removers[1]. However, these techniques have the potential to remove the graphene layer simultaneously. The remover PG (an NMP-based solvent[2]) can dissolve moderately cross-linked SU-8 at $80^{\circ}C$ or less and is used in this work. The trade-off between the enhanced adhesion of highly cross-linked SU-8 to graphene (and the substrate) and the lower probability of removing more cross-linked SU-8 from the substrate emphasizes the importance of optimizing steps of lithography process.

PG at room temperature, PG at $50^{\circ}C$, and the NMP solvent were used in the wet etching process during the different tests. PG is a solvent stripper[2], and wet etching of the SU-8 pattern is usually performed by peeling the SU-8 pattern off rather than completely solving the SU-8 pattern. SU-8 quickly saturates PG and NMP in the first bath, requiring a second or larger bath. The same results were obtained for the three solvents, but the stripping of SU-8 by PG, especially PG at $50^{\circ}C$, was faster. Fig. 2(a) (in main text) shows a microscopy image of the SU-8 strips, which peel from the substrate in the bath of PG remover while creating channels in the graphene layer.

The SU-8 pattern is simpler to remove mechanically, especially if the SU-8 elements are thick, closely spaced, and interconnected. In the mechanical removal method, one piece of Scotch tape covers the SU-8 pattern, and then, by peeling the Scotch tape off, this pattern and all the graphene parts connected to it are removed. In the microscopic images of Fig. 2(b) (in main text), the SU-8 strips are being peeled off from the substrate using Scotch tape (from Graphene Supermarket Company[3]) and creating channels on the graphene layer.

The first problem with the wet etching method occurs in extensive SU-8 patterns, as shown in Fig. 2. In these cases, some parts of the SU-8 pattern separate earlier than others do. These detached parts can move with the solvent flow, collide with the graphene layer, and cause damage to it. The second problem is the possible redeposition of peeled-off SU-8 elements, which remain as contaminants in the final sample. Therefore, if wet etching is chosen, some arrangements are required to remove the separated SU-8 parts via solvent flow. In the clean and accurate mechanical removal method, the degree of SU-8 contamination is lower. All the contamination could be completely removed by the proposed method, which is compatible with imprint lithography (fifth section in main text). Owing to its simplicity and low degree of contamination, the graphene layer in most samples in this study was removed via a mechanical removal method.

S2:

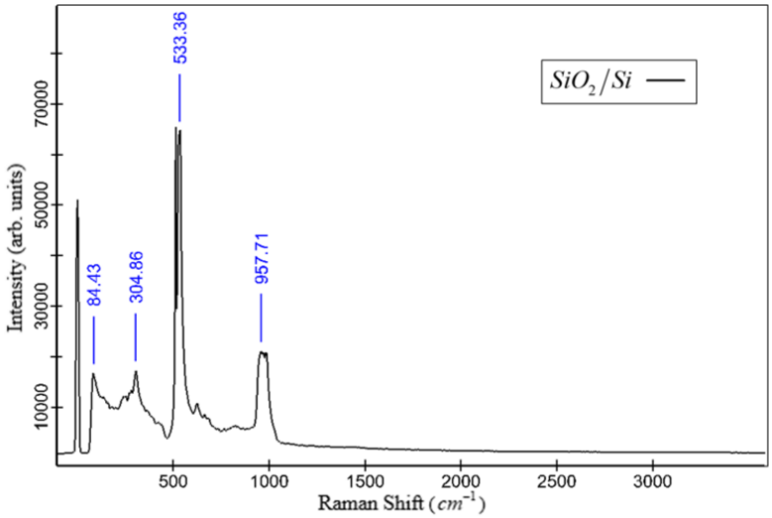

Figure S1. The Raman spectrum includes characteristic peaks at a random point in the SiO_2/Si substrate without any coating.

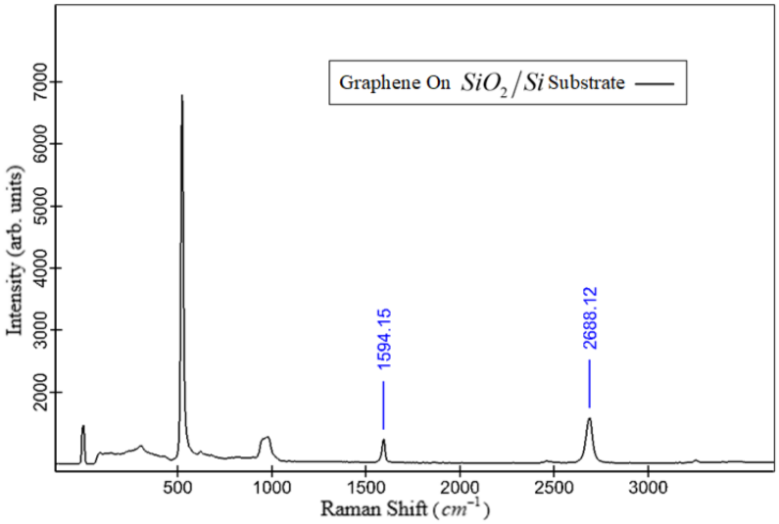

Figure S2. The Raman spectrum includes characteristic peaks at a random point in the graphene layer on the SiO_2/Si substrate. This spectrum does not show a *D* peak.

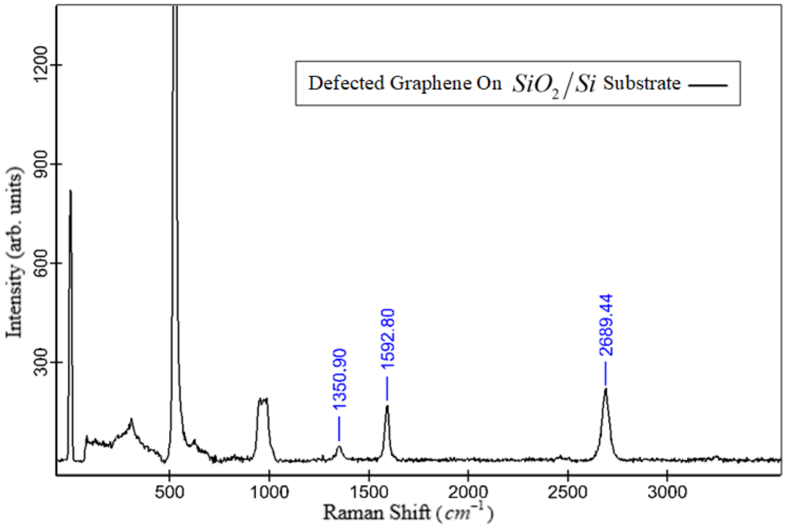

Figure S3. The Raman spectrum includes characteristic peaks at a random point in the graphene layer on the SiO_2/Si substrate. This spectrum shows a D peak.

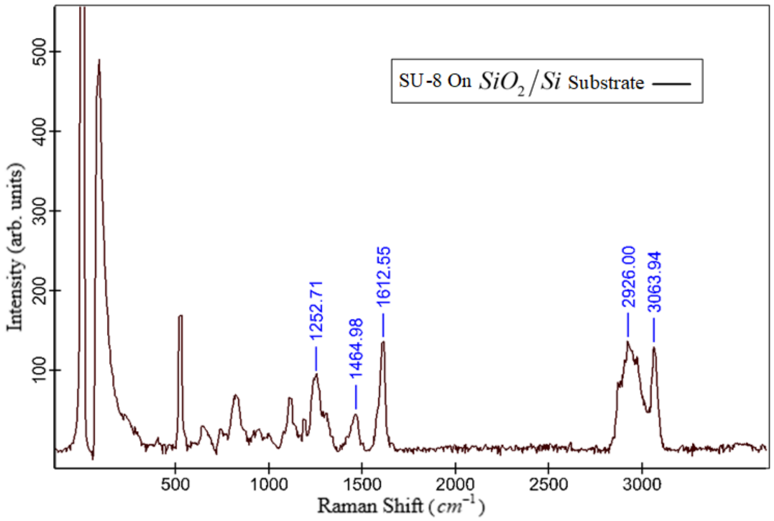

Figure S4. The Raman spectrum includes characteristic peaks at a random point in the SU-8 layer on the SiO_2/Si substrate.

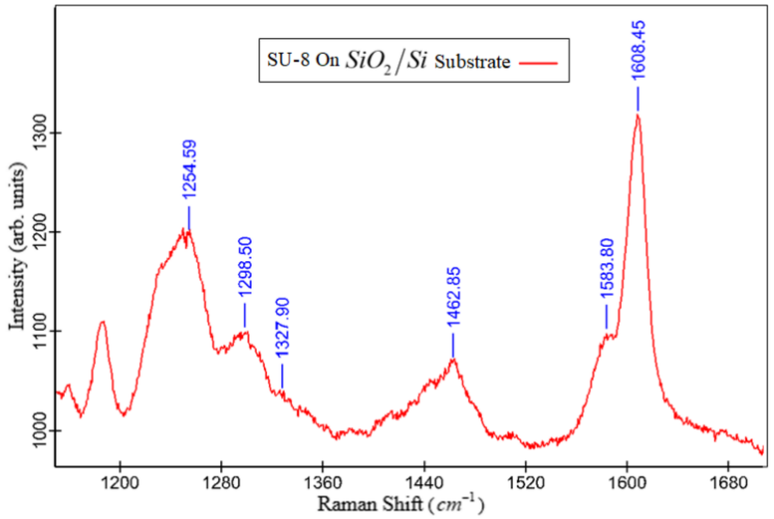

Figure S5. The Raman spectrum includes characteristic peaks at a random point in the SU-8 layer on the SiO_2/Si substrate. A spectrometer with a 1200 grooves/mm was used to measure the Raman spectrum. There is a shoulder on the SU-8 peak around 1584 cm^{-1}.

S3:

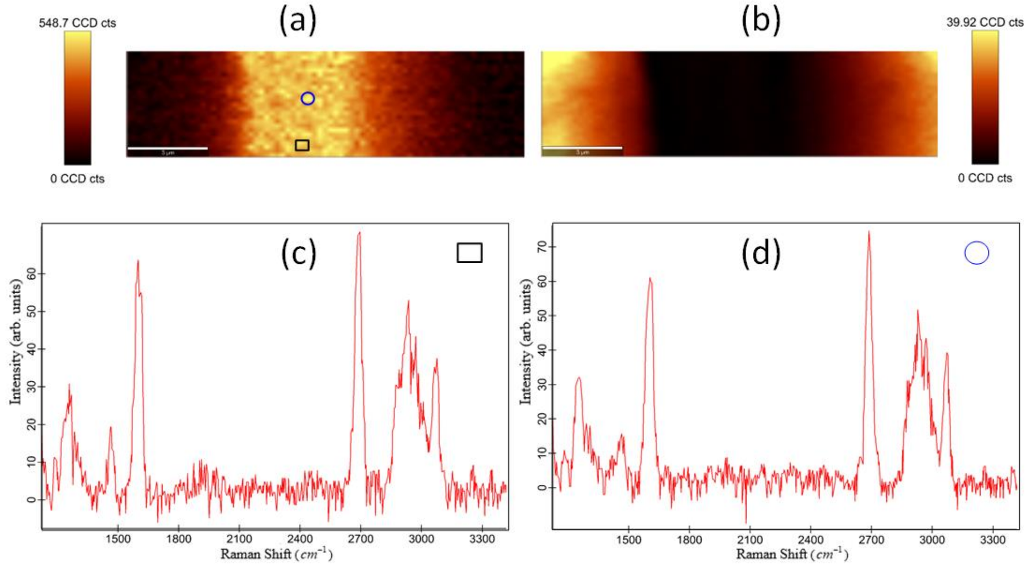

Figure S6. The $2667 Cm^{-1}$ and $2D$ Raman intensity maps of a sample with a $5 \mu m$ SU-8 strip on the graphene layer are visible in (a) and **(b),** respectively. The Raman spectra of the two random points at the center of the rectangle and circle are shown in **(c)** and (d). The G and D peaks of graphene are not obvious because of the more intense peaks of SU-8. In this measurement, the Raman excitation laser was focused on the graphene surface outside the SU-8 strip. The scale bar in **(a)** and **(b)** is $2 \mu m$. The color scale bar shows the different intensities in each map. In the color bar, two number bounds are selected to provide the greatest possible contrast in the image.

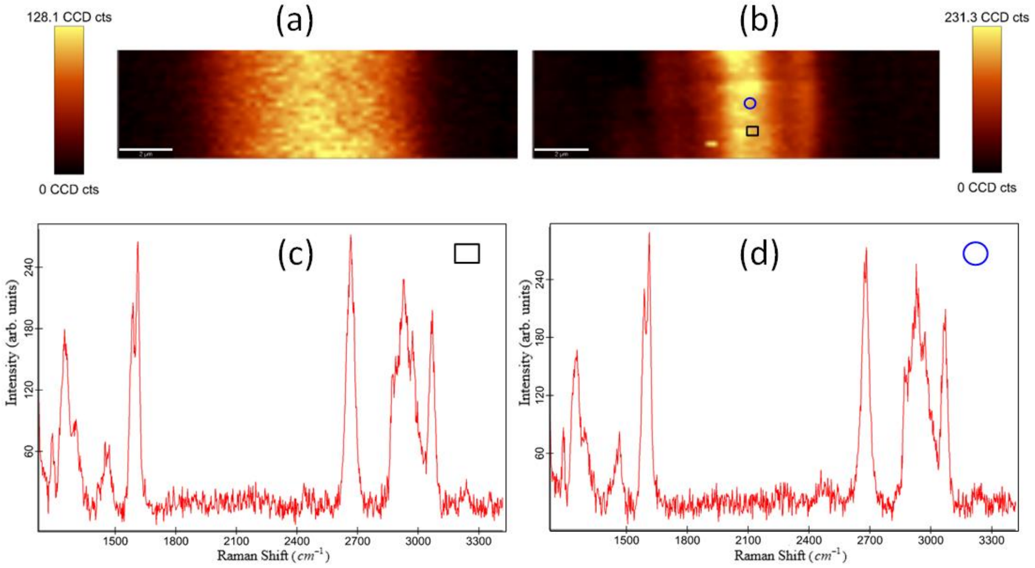

Figure S7. The $2667Cm^{-1}$ and *2D* Raman intensity maps of a sample with a $5\mu m$ SU-8 strip on the graphene layer are visible in **(a)** and **(b),** respectively. The Raman spectra of the two random points at the center of the rectangle and circle are shown in **(c)** and **(d)**. The *D* peak of graphene is not obvious because of the more intense peaks of SU-8. In this measurement, the Raman excitation laser was focused on the graphene surface under the SU-8 strip. The scale bar in **(a)** and **(b)** is $2\mu m$. The color scale bar shows the different intensities in each map. In the color bar, two number bounds are selected to provide the greatest possible contrast in the image.

S4:

The presence of the SU-8 strip in this sample facilitates the detection of points exhibiting even minimal SU-8 peak intensities. This allows for the identification of any remaining SU-8 contamination within the sample.

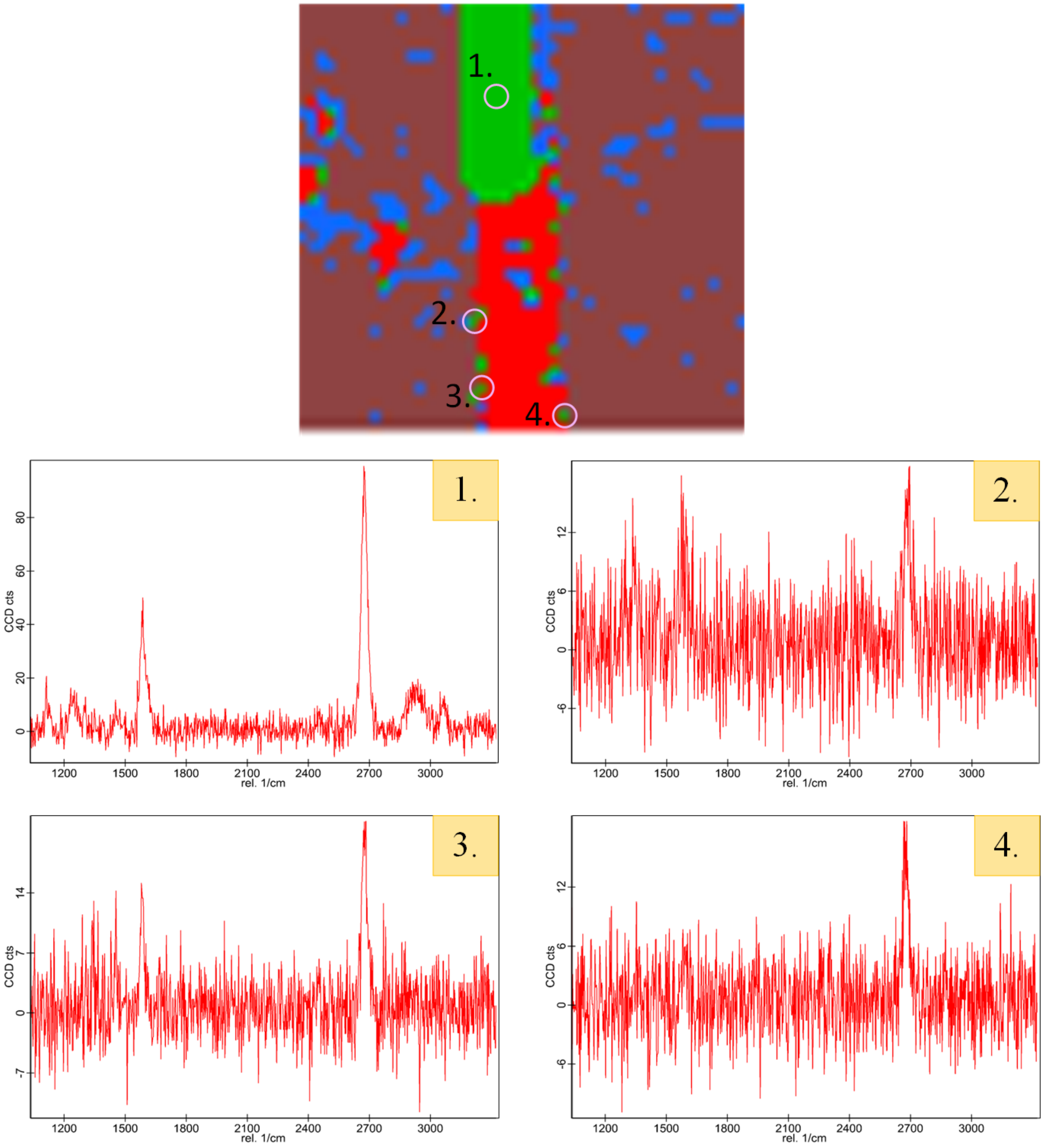

Figure S8: A cluster image of Fig. 5(c) from the main text is shown. Single Raman spectra at four points of SU-8 contamination, located at the centers of the pink circles, are displayed in plots 1 to 4. The numbers on the vertical axis indicate the low peak intensities of points 2, 3, and 4 relatives to point 1.

S5:

The number of graphene layers is determined in different ways, such as X-ray photoelectron spectroscopy (XPS), angle-resolved ultra-violet photoemission spectroscopy (ARUPS), low-energy electron diffraction (LEED), and Raman spectroscopy [4]. Raman spectroscopy has been more frequently used in the literature [4-6]. According to the stacking order of graphene layers and possible bonds between them, there are different patterns to define the number of graphene layers. CVD graphene has a local stacking order, which is different from Bernal stacking [4,5,7]. Although the *2D* peak line shape and linewidth clearly identify the few numbers of layers in a Bernal graphene layer, additional data is required to determine the number of CVD graphene layers. For example, the linewidth of the Lorentzian *2D* peaks of both monolayer and bilayer CVD graphene was reported in the interval of $30-83\ Cm^{-1}$ [4,6]. Some proper information is the *2D* peak to *G* peak intensity ratio (I_{2D}/I_G), the presence of *C* modes, LMB modes, some characteristic peaks adjacent to the *G* peak, or the presence of a $D' \pm ZO'$ peak [4,5].

In the current paper, we used the *2D* peak to *G* peak intensity ratio (I_{2D}/I_G), *2D* peak linewidth, and *2D* peak line shape to identify the number of graphene layers on the SiO_2/Si substrate. For this goal, Raman spectra were taken in several areas of different samples before any process. In each case, hundreds of Raman spectra were obtained by raster scanning the laser within an area, which is called the Raman map. Using the Raman map, the I_{2D}/I_G map was calculated (Figure S8(a)).

The refractive index and thickness of the optical media surrounding graphene (such as substrate) as well as the Raman laser wavelength affect I_{2D}/I_G [8]. Different values of I_{2D}/I_G for monolayer, bilayer, or multilayer graphene have been reported with different substrates and Raman laser wavelengths [4,6]. In this paper, graphene layers on SiO_2/Si substrates with a $285nm$ SiO_2 thickness and a Raman laser wavelength of $532nm$ were used. The value of I_{2D}/I_G for monolayer, bilayer, and multilayer graphene with these conditions has already been published by Li *et al.* [8].

The I_{2D}/I_G map for one area of a sample is shown in Figure S8. The Raman spectrum of each point at the center of the three circles on this map is shown in Figure S8(a), S8(b), and S8(c). The Lorentzian *2D* peak in Figure S8(a) with $FWHM = 37cm^{-1}$ and $I_{2D}/I_G = 2$ shows a monolayer graphene in point (a). The Lorentzian *2D* peak in Figure S8(b) with $FWHM = 42cm^{-1}$ and $I_{2D}/I_G = 1.3$ shows a bilayer graphene in point (b), and finally, the *2D* peak in Figure S8(c) with $FWHM = 45cm^{-1}$ and $I_{2D}/I_G = 0.59$ shows a multilayer graphene in point (c). These values are consistent with the values reported by Li *et al.* [8]. Investigation of different areas of samples revealed that multilayer graphene was present in just small areas of our samples.

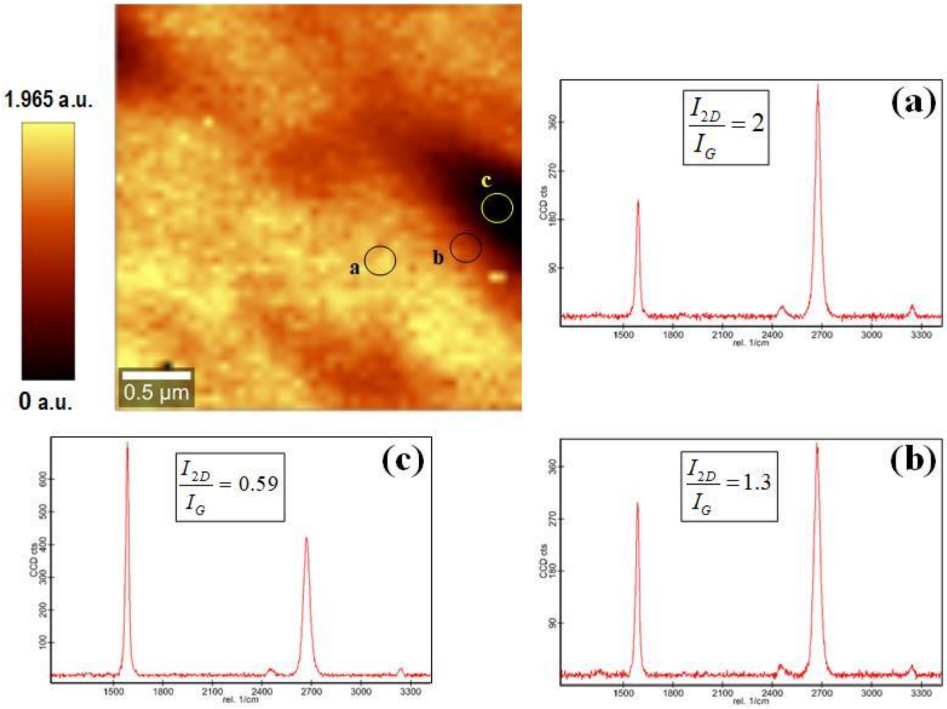

Figure S9. I_{2D}/I_G map for one area of a sample. The Raman spectra of three points in the center of three circles were shown in **(a)**, **(b)**, and **(c)**. The ratio of I_{2D}/I_G , *2D* linewidth, and *2D* line shape reveal that graphene is monolayer in point **(a)**, bilayer in point **(b)**, and multilayer in point **(c)**. The various amounts of I_{2D}/I_G have been displayed by the color scale bar. In the color bar, two number bounds are selected to provide the greatest possible contrast in the image.

S6:

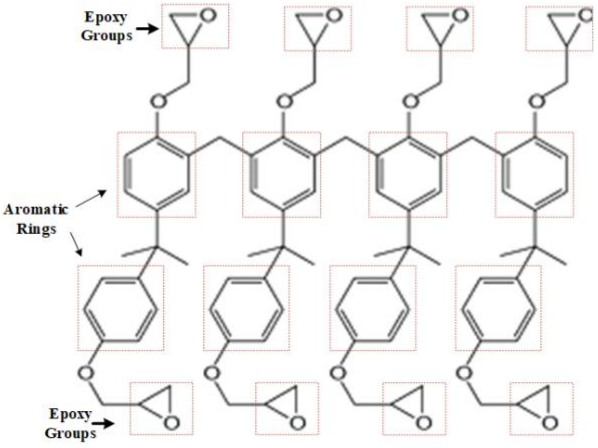

Figure S10. SU-8 monomer with eight epoxy rings and aromatic rings has been shown.

S7:

The initial samples of the graphene layer on the SiO_2/Si substrate usually display spatial inhomogeneity for Raman peak frequencies and widths [9,10]. Different parts of the graphene layer with different initial properties experience different values of extra strain and doping during the lithography process. So, the same change in the Raman spectra during the lithography process is not expected for all points of the graphene layer. Therefore, some fixed points on the graphene layer must be selected for investigation during the lithography process. It is also worth mentioning that the Raman spectrum of each fixed point is affected by its surrounding points within the laser spot area [11,12].

The microscopic images and Raman spectra were investigated at some fixed points of graphene on SiO_2/Si substrate in the initial step, after SU-8 coating and SB as the second phase, and after UV exposure and PEB as the third phase. The developing step (the sixth step in Figure 1) was ignored to easily find the fixed points in the next step. In order to repeatedly locate the fixed point with the microscope after each step, a precise path on the sample to find the fixed point was recorded. The image of the fixed point was also recorded with 10X, 20X, 50X, and finally 100X objectives with some key marks.

S8:

At a few fixed points, the change of the Raman spectra during the lithography process was examined. The *G* and *2D* properties for four fixed points are shown in the following tables. The first row of tables demonstrates the extracted data of the initial Raman spectra of the graphene on the SiO_2/Si substrate before any process (the initial step). The rows labeled "Coat & S.B." belong to the spectra after SU-8 coting and soft baking. The rows labeled "UV and PEB" contain fixed point data after UV exposure and post-exposure baking.

Due to the fact that finding fixed points was time-consuming, approximately one to three hours after each baking step, the Raman spectra were measured. In the last Raman measurement, the fixed points were subjected to another run of Raman spectroscopy one day following the post-exposure bake. After a day, some details of the Raman spectra varied at some points and remained constant at others. So, the environmental conditions may change the adhesion of graphene and SU-8, especially over a longer time interval. The extracted data from this step is visible in the last row of the tables (after 1 day).

Table S1. Extracted data of the *G* and *2D* peaks of the Raman spectra at the first fixed point.

First Point	ω_G		FWHM-*G*		ω_{2D}		FWHM-*2D*	
	Value	Error	Value	Error	Value	Error	Value	Error
Initial Step	1604.00939	0.1382	16.04003	0.45565	2695.82169	0.12304	36.50765	0.4665
Coat & S.B.	1599.60513	0.17126	20.88523	0.58332	2692.00838	0.16777	35.46836	0.62948
UV&PEB	1600.30266	0.26995	19.30294	0.89716	2692.34313	0.28043	38.81669	1.08416

after1day	1602.913	0.19983	18.7677	0.66717	2695.673	0.20883	36.84993	0.79417

Table S2. Extracted data of the *G* and *2D* peaks of the Raman spectra at the second fixed point.

Second Point	ω_G		FWHM-*G*		ω_{2D}		FWHM-*2D*	
	Value	Error	Value	Error	Value	Error	Value	Error
Initial Step	1596.8803	0.1180	16.9949	0.4069	2687.6732	0.1021	32.4702	0.3943
Coat & S.B.	1600.1801	0.1602	20.4170	0.5772	2693.5885	0.1540	36.7979	0.6055
UV&PEB	1599.7237	0.2064	18.0155	0.6940	2691.5242	0.2371	36.3307	0.9272
after1day	1598.76635	0.20689	18.13132	0.74715	2691.5994	0.18325	34.5004	0.7038

Table S3. Extracted data of the *G* and *2D* peaks of the Raman spectra at the third fixed point.

Third Point	ω_G		FWHM-G		ω_{2D}		FWHM-2D	
	Value	Error	Value	Error	Value	Error	Value	Error
Initial Step	1598.76445	0.12128	16.25054	0.41711	2690.14134	0.10153	34.21884	0.38853
Coat & S.B.	1595.23004	0.23214	18.85098	0.90748	2689.10764	0.19265	35.13349	0.78305
UV&PEB1	1598.99675	0.15692	17.97184	0.56102	2692.21971	0.1935	35.92515	0.69869
after1day	1597.54891	0.22858	18.7093	0.83336	2692.19327	0.20802	33.99268	0.78297

Table S4. Extracted data of the *G* and *2D* peaks of the Raman spectra at the fourth fixed point. At this point, Raman spectra were not measured after SU-8 coating and soft baking.

Fourth Point	ω_G		FWHM-*G*		ω_{2D}		FWHM-*2D*	
	Value	Error	Value	Error	Value	Error	Value	Error
Initial Step	1600.94319	0.13713	16.22585	0.4787	2693.37346	0.15734	37.20673	0.62139
UV&PEB	1602.57078	0.16621	18.61149	0.58039	2697.26114	0.22392	37.59741	0.85403
after1day	1601.79969	0.1971	18.21761	0.67792	2695.00062	0.20389	36.73369	0.80202

S9:

The investigation shows that the Raman spectra of the fixed points, which did not have a visible *D* peak in the initial steps, do not also have a *D* peak after the $600nm$ SU-8 coating and the lithography process. In reality, not a single one of the fitted subpeaks agrees with a *D* peak. A very small *D* peak can sometimes be fitted due to the noise in the

spectrum, but its area and height standard errors are significantly larger than their main values. These peaks were thus dismissed (Figure S10).

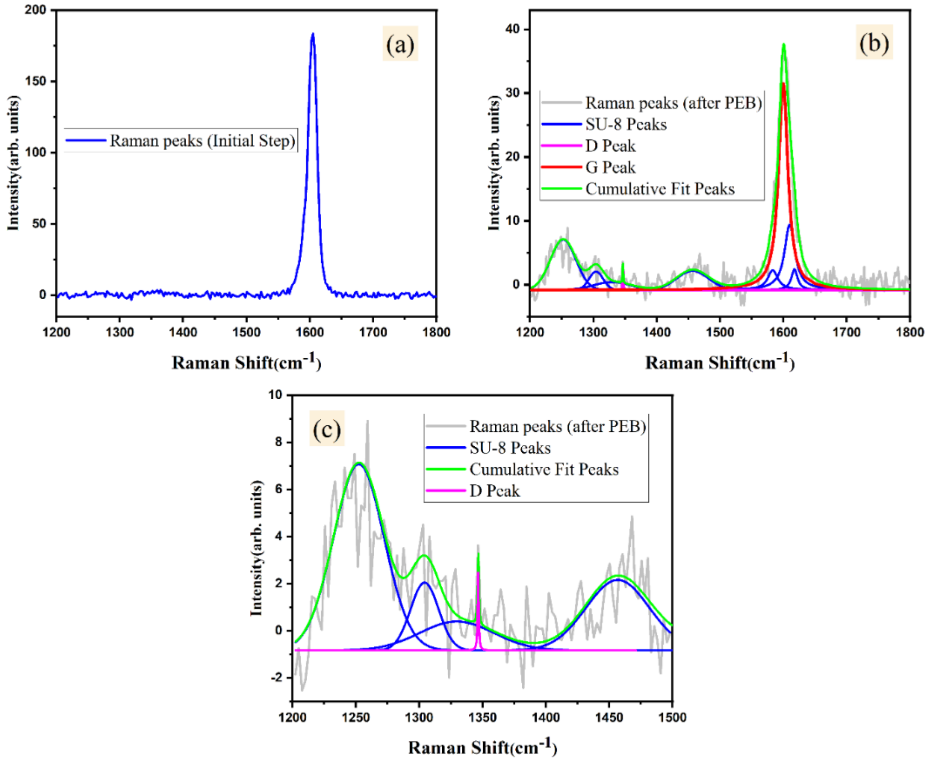

Figure S11. **(a)** The Raman spectrum of the first fixed point on the graphene layer before any processes, which does not have a *D* peak. **(b)** The Raman spectrum of this point after the UV exposure and PEB steps was fitted with the blue subpeaks of SU-8, the red *G* peak of graphene, and one unacceptable subpeak at $1350\ Cm^{-1}$. The range $1200-1500 cm^{-1}$ of this spectrum in **(c)** clearly shows the unacceptable pink subpeak. The unacceptable pink subpeak has the following characteristics: $\omega = 146.61, \Delta\omega = 11.84$, $Area = 5.19, \Delta Area = 33.01$, $Height = 3.30, \Delta Height = 39.40$. In the analysis, the limit of 1 was considered for the height. The adaptation of the pink subpeak to noise and the fact that its parameters have error values higher than the main values suggest that this subpeak is unacceptable.

S10:

Comparison of AFM results across samples indicates that graphene surface roughness remains almost consistent before and after patterning graphene, except at the edges of the final graphene pattern. The variations in surface roughness along multiple lines are presented in the AFM measurements of the samples (Figures S7, S8, S9, and S10). The surface roughness of graphene, away from the edges, is approximately 2 nm. The height value of the edge of the final graphene pattern depends on some factors, such as the strength of the graphene adhesion to SiO_2/Si , the characteristics of the bordering graphene points, and the amount and direction of scotch tape movement used for SU-8 peeling off (Figure S11). This height was found to be less than $25nm$ at

most points on the edges in several samples. However, in some samples, it reached a huge height of about $300nm$ at individual points. The cause of this excessive edge height was often pollution. The edge height of graphene in Figure S12 illustrates this problem with the AFM measurement.

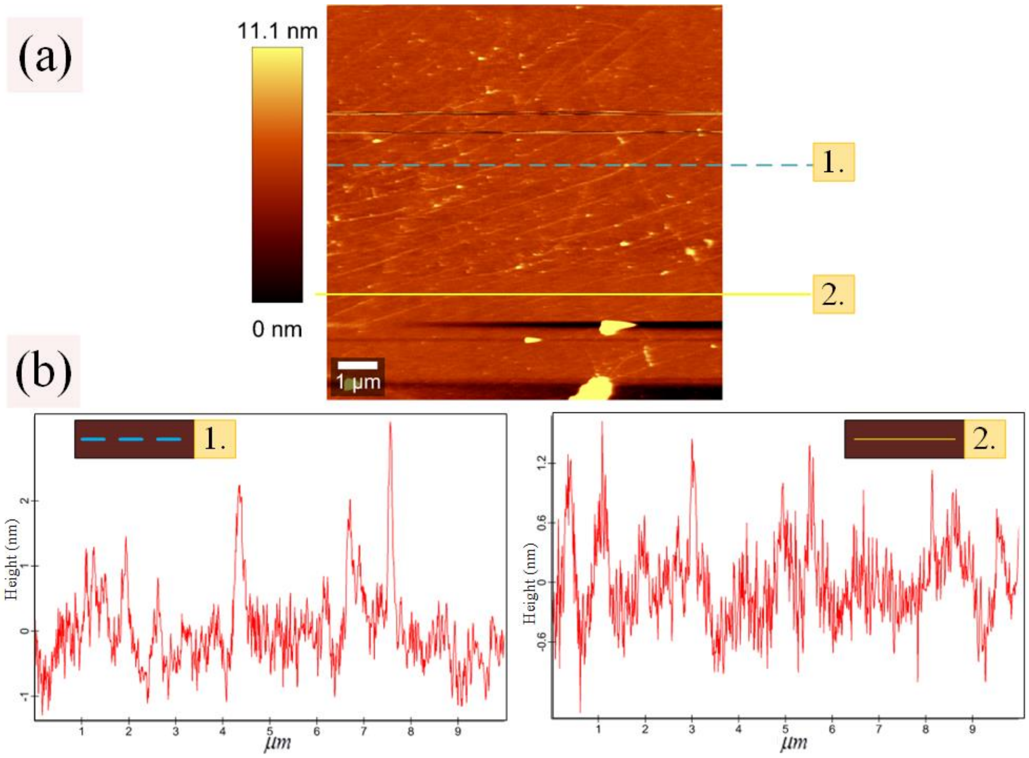

Figure S12. (a) An AFM topography image of an initial graphene sample resulted in AC mode. The color scale bar represents height variations across the sample, with two bounds selected to maximize contrast in the image. **(b)** Cross-section profiles of the surface topography along two lines. The cross-section of the topography image along the blue and yellow lines (lines 1 and 2) illustrates the surface roughness of the initial graphene before the lithography process.

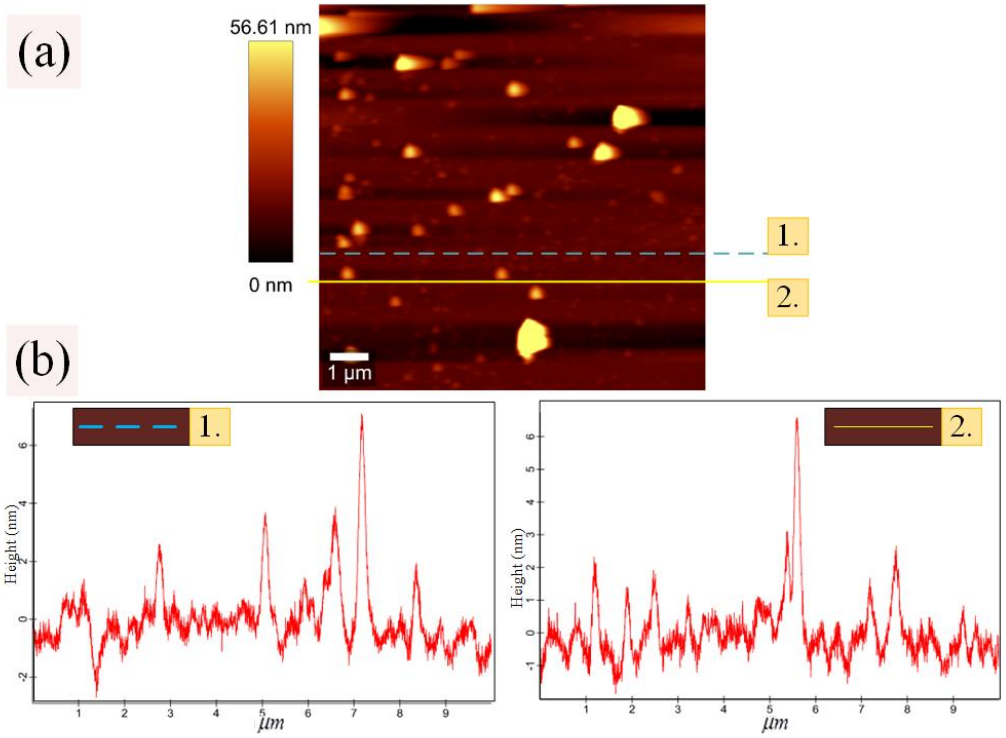

Figure S13. **(a)** An AFM topography image of an initial graphene sample, with surface contamination, in AC mode. The color scale bar represents height variations across the sample, with two bounds selected to maximize contrast in the image. **(b)** Cross-section profiles of the surface topography along two lines. The cross-section of the topography image along the blue and yellow lines (lines 1 and 2) illustrates the surface roughness of the initial graphene before the lithography process.

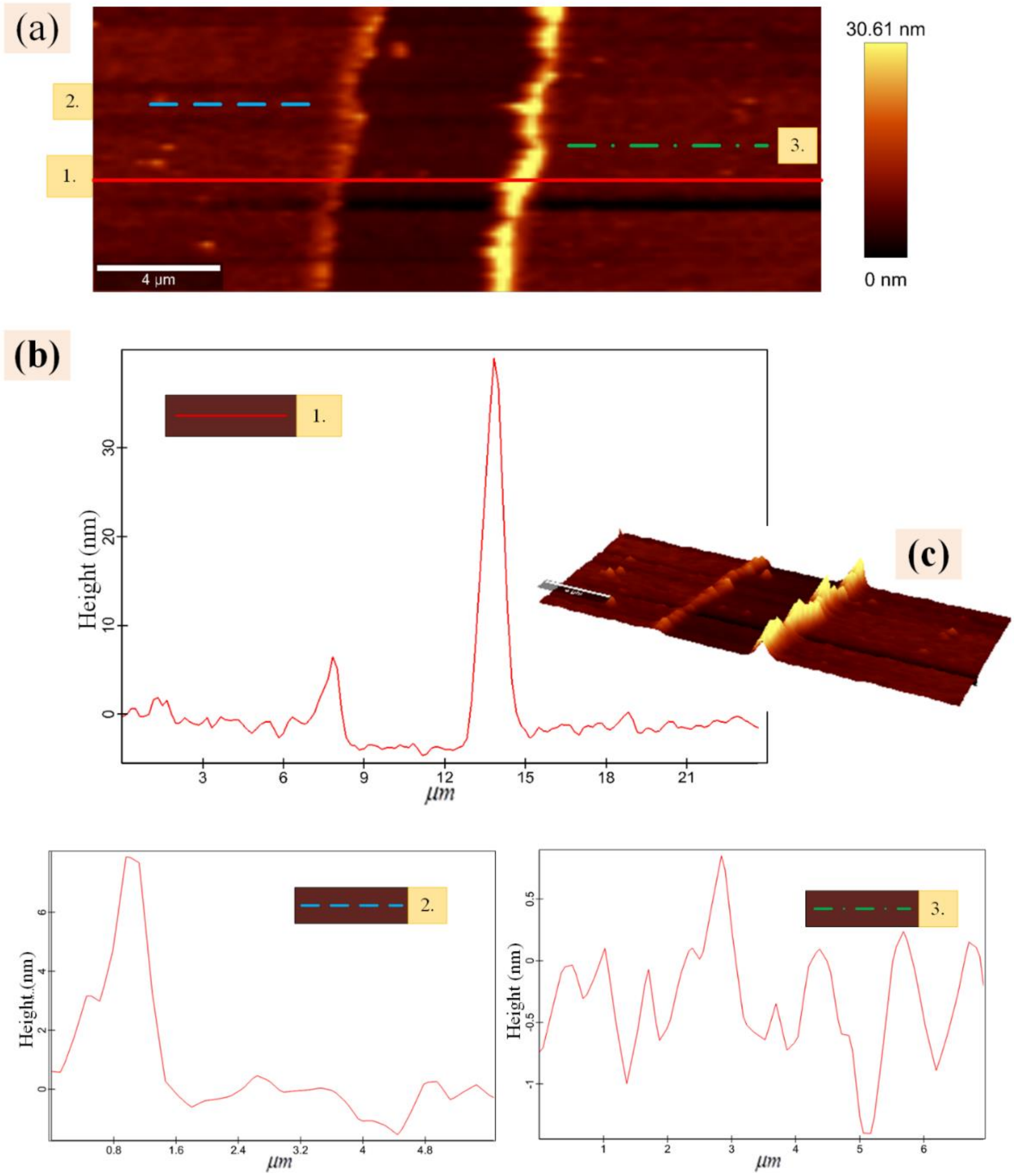

Figure S14. This image shows the surface topography of one graphene sample measured by AFM in AC mode **(a)** in two-dimensional vision and **(c)** in three-dimensional vision. In this sample, lithography and the removal of the cross-linked SU-8 strip have created a channel on the graphene layer. The various heights in the sample are displayed by the color scale bar. It is clear that one of the graphene channel sides has a higher height than the other, and this side corresponds with the direction in which the SU-8 strip has been peeled off. For more clarity, the cross-section of the topography image along the red line is shown at the first curve in **(b)**. Also, the cross-section of the topography image along the blue and green short lines (lines 2 and 3) illustrates the surface roughness of the graphene in regions away from the edge.

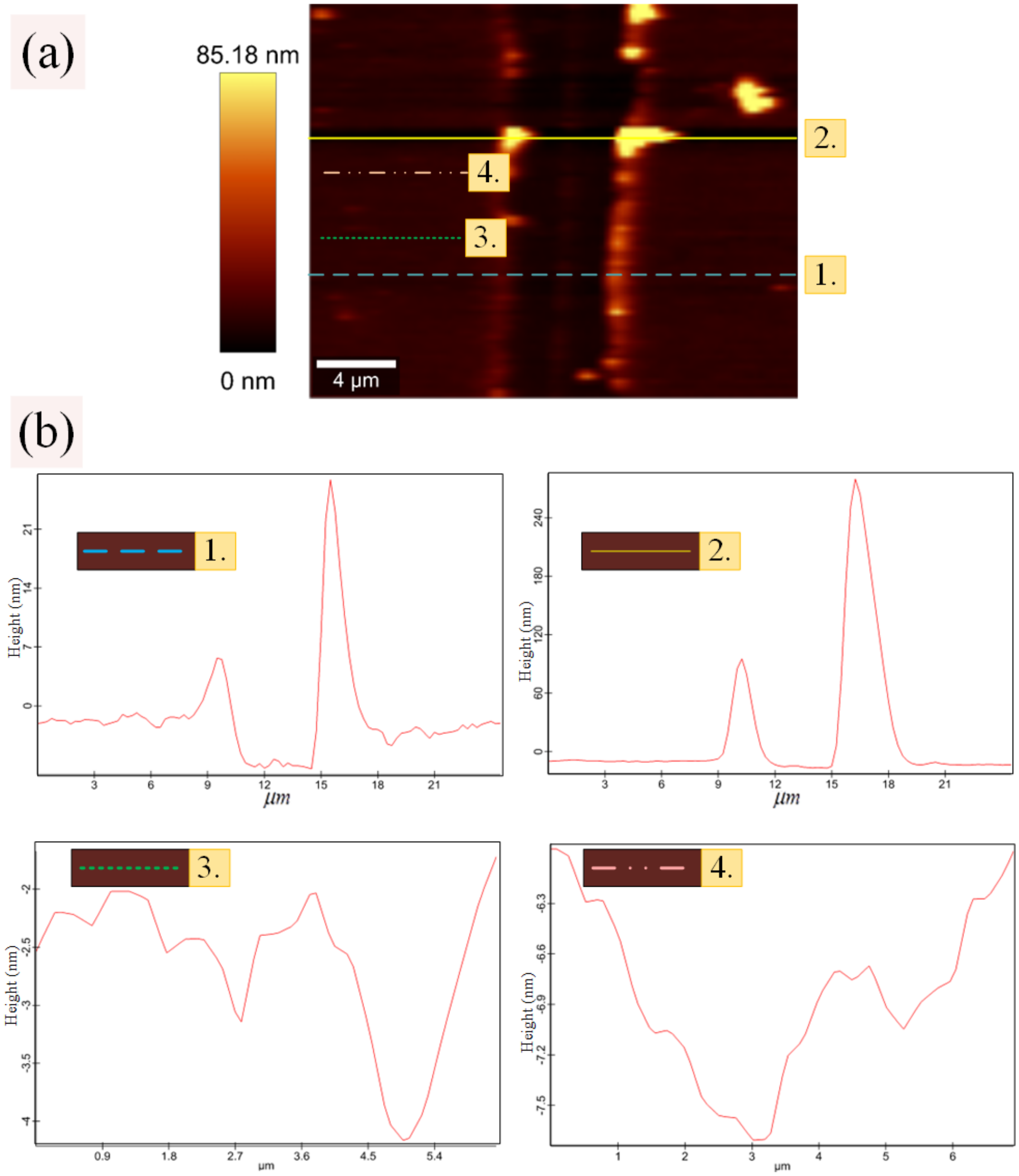

Figure S15. (a) An AFM topography image of a graphene sample resulted in AC mode. In this sample, lithography and the removal of the cross-linked SU-8 strip have created a channel on the graphene layer. The color scale bar represents height variations across the sample, with two bounds selected to maximize contrast in the image. **(b)** Cross-section profiles of the surface topography along four lines. The yellow solid line passes through the highest point, SU-8 residue, at the graphene edge (line 2). The blue dashed line represents a typical height at other locations along the graphene edge. The pink and green short lines (lines 3 and 4) illustrate the surface roughness of the graphene in regions away from the edge.

References:

1 Kim, B. J. & Meng, E. Review of polymer MEMS micromachining. *Journal of Micromechanics and Microengineering* **26**, 013001 (2015).

2 *Remover PG*, <https://kayakuam.com/wp-content/uploads/2020/11/KAM-Remover-PG-TDS.10.29.20-final.pdf> (

3 *Graphene Supermarket Company*, <https://www.graphene-supermarket.com/> (

4 Malard, L., Pimenta, M. A., Dresselhaus, G. & Dresselhaus, M. Raman spectroscopy in graphene. *Physics reports* **473**, 51-87 (2009).

5 Ferrari, A. C. & Basko, D. M. Raman spectroscopy as a versatile tool for studying the properties of graphene. *Nature nanotechnology* **8**, 235-246 (2013).

6 Reina, A. *et al.* Large area, few-layer graphene films on arbitrary substrates by chemical vapor deposition. *Nano letters* **9**, 30-35 (2009).

7 Dimiev, A. *et al.* Layer-by-layer removal of graphene for device patterning. *Science* **331**, 1168-1172 (2011).

8 Li, X. *et al.* Large-area synthesis of high-quality and uniform graphene films on copper foils. *science* **324**, 1312-1314 (2009).

9 Lee, J. E., Ahn, G., Shim, J., Lee, Y. S. & Ryu, S. Optical separation of mechanical strain from charge doping in graphene. *Nature communications* **3**, 1-8 (2012).

10 Olziersky, A. *et al.* Insight on the SU-8 resist as passivation layer for transparent Ga 2 O 3–In 2 O 3–ZnO thin-film transistors. *Journal of Applied Physics* **108**, 064505 (2010).

11 Beams, R., Cançado, L. G. & Novotny, L. Raman characterization of defects and dopants in graphene. *Journal of Physics: Condensed Matter* **27**, 083002 (2015).

12 Neumann, C. *et al.* Raman spectroscopy as probe of nanometre-scale strain variations in graphene. *Nature communications* **6**, 8429 (2015).
